# Laser Safety Calculations for Imaging Sensors

**DOI:** 10.3390/s19173765

**Published:** 2019-08-30

**Authors:** Gunnar Ritt

**Affiliations:** Fraunhofer IOSB, Gutleuthausstr. 1, 76275 Ettlingen, Germany; gunnar.ritt@iosb.fraunhofer.de

**Keywords:** laser safety, laser dazzle, laser damage, imaging sensors

## Abstract

This publication presents an approach to adapt the well-known classical eye-related concept of laser safety calculations on camera sensors as general as possible. The difficulty in this approach is that sensors, in contrast to the human eye, consist of a variety of combinations of optics and detectors. Laser safety calculations related to the human eye target terms like Maximum Permissible Exposure (MPE) and Nominal Ocular Hazard Distance (NOHD). The MPE describes the maximum allowed level of irradiation at the cornea of the eye to keep the eye safe from damage. The hazard distance corresponding to the MPE is called NOHD. Recently, a laser safety framework regarding the case of human eye dazzling was suggested. For laser eye dazzle, the quantities Maximum Dazzle Exposure (MDE) and the corresponding hazard distance Nominal Ocular Dazzle Distance (NODD) were introduced. Here, an approach is presented to extend laser safety calculations to camera sensors in an analogous way. The main objective thereby was to establish closed-form equations that are as simple as possible to allow also non-expert users to perform such calculations. This is the first time that such investigations have been carried out for this purpose.

## 1. Introduction

Laser dazzle is a topic that has gained more and more attraction during the last years. The reason might be the increasing proliferation of high-power laser pointers and the associated misuse of these devices, which is well documented in aviation [1,2]. Besides the misuse of lasers in the civilian area, the military uses laser devices as an optical countermeasure [3]. Laser dazzlers are developed to dazzle humans but also sensor systems [4]. Such laser systems pose a threat for camera systems (e.g., surveillance cameras or cameras used in unmanned vehicles), since they are highly susceptible to laser illumination. Since laser devices are offered with nearly any possible wavelength in the visible spectral range, the protection against such threats is not possible using classical laser eye protection. Classical laser protection filters are based on absorption or interference and their operating range is limited to specific wavelengths or wavelength bands. Therefore, some research of the last years was dedicated to wavelength-independent or tunable protection measures, like liquid crystal Lyot filters [5], augmented reality headsets [6] or the use of pupil-plane phase elements [7,8]. Our own research on laser dazzle protection is focused on the development of an active laser light suppression concept based on the use of a digital micromirror device (DMD) in combination with wavelength multiplexing [9,10]. Another approach is the use of complementary wavelength bands in optical sensors to avoid image information lost in case of laser dazzle [11]. Laser dazzling of sensors was intensively studied experimentally and theoretically by various groups [12,13,14,15,16,17,18], including approaches to quantify the performance of protection measures [19,20].

**Figure 1:**
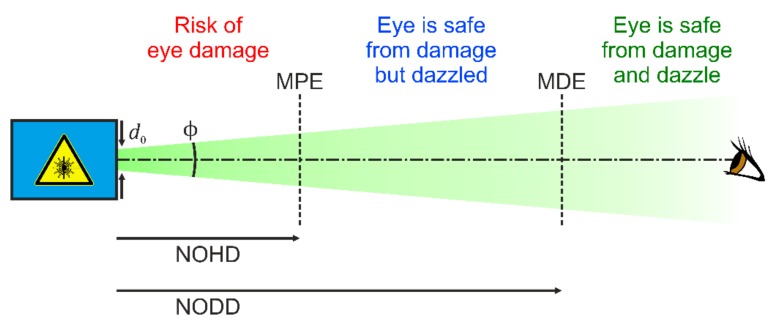
Hazard distances for the human eye: Nominal Ocular Hazard Distance (NOHD) and Nominal Ocular Dazzle Distance (NODD). MPE: Maximum Permissible Exposure; MDE: Maximum Dazzle Exposure.

A lot of research was also done regarding laser dazzle of the human eye. This includes the modelling of laser eye dazzle [21,22] and the investigation of degradation of human performance in laser dazzle situations [23,24]. A highlight might be the work of Williamson and McLin, which have extended the traditional laser safety quantities of Maximum Permissible Exposure (MPE) and Nominal Ocular Hazard Distance (NOHD) to laser dazzle [25,26]. MPE and NOHD refer only to laser damage of the human eye. MPE is the maximum laser irradiance at the cornea of the eye that ensures safety from damage when looking into the direct laser beam. The distance to a laser source at which the laser irradiance dropped to the MPE, is called NOHD. This is illustrated in Figure 1.

Initially, with the help of human observer trials, McLin and co-workers adapted the CIE equations for disability glare based on broadband light sources to laser radiation [27]. Then, Williamson and McLin set up a framework to determine the Maximum Dazzle Exposure (MDE) and the corresponding hazard distance, named the Nominal Ocular Dazzle Distance (NODD) [25,26]. These new quantities are also included in Figure 1. In contrast to the quantity MPE accounting for eye damage, which depends mainly on laser wavelength and exposure duration, the dazzle quantity MDE depends on more parameters: age and pigmentation of the human eye, target size and contrast, and background luminance. In their framework it is proposed to calculate MDE values for three ambient luminance levels (approximating light conditions at night, dusk/dawn and day) and four dazzle levels (corresponding to different visual obscuration fields: 2°, 10°, 20°, and 40°) [26].

Since we work intensively on laser dazzle protection of camera sensors at our institute, the question arose: Is it possible to extended laser safety calculations to imaging sensors? Equivalent to laser safety calculations for the human eye, calculations for sensors should include:

1. The statement of a maximum laser irradiance to prevent sensor damage:
  *Maximum Permissible Exposure for a Sensor*, MPE_S_
2. Statement of a hazard distance corresponding to the MPE_S_:
  *Nominal Sensor Hazard Distance*, NSeHD
3. Statement of a laser irradiance that corresponds to a certain dazzle level:
  *Maximum Dazzle Exposure for a Sensor*, MDE_S_
4. Statement of a hazard distance corresponding to the MDE_S_:
  *Nominal Sensor Dazzle Distance*, NSeDD
5. The possibility to calculate the size of a dazzle spot at the imaging sensor depending on the parameters of laser source, camera lens and imaging sensor.

For the exposure quantities, the subscript “S” is used to distinguish the quantities for sensors from those for the human eye. For the hazard distances, the capital “O” (for “Ocular”) is replaced by “Se” (for “Sensor”) to distinguish the sensor quantities from those for the human eye. The use of a simple “S” for the replacement is not possible, because the abbreviation NSHD is already in use for the Nominal Skin Hazard Distance.

The aim is to set up equations to perform such calculations with the following constraints:

1. Equivalent to laser safety calculations for the human eye, the values of MPE_S_ and MDE_S_ shall be stated for the position of the entrance aperture of the camera lens. In this case, a user can position a power meter at a well-accessible place to compare calculated exposure values with the laser irradiance.
2. Since users, who are not experts in the field, should also be able to be perform such calculations, closed-form expressions shall be derived containing only well-known operations and functions. The equations should be as simple as possible but still sufficiently accurate. In any case, the necessity of performing calculations using a computer should be avoided because of equations that can only be solved numerically.
3. As far as possible, the equations shall include only standard parameters that are usually specified by the manufacturer of laser source, camera lens or imaging sensor.

Threshold values for the laser-induced damage (LIDT) of imaging sensors are known, for example see the work of Schwarz et al. [28], but usually these threshold values are related to the imaging sensors located at the focal plane of the camera lens and not to the front side of the camera lens. If we want to fulfil the first constraint, we have to transfer the focal plane damage threshold to a corresponding value at the position in front of the camera lens. This can only be accomplished if the light distribution at the imaging sensor, and in particular the focal plane peak irradiance, can be described quantitatively by the parameters of the incident laser light and the camera lens.

The same statement is valid for laser dazzle. A laser dazzle threshold can be defined, for example, as the irradiance, where the pixels of the imaging sensor start to saturate. Such saturation thresholds can be calculated easily from the specifications of the imaging sensor [10]. However, such threshold values again only apply to the location of the imaging sensor at the focal plane. As before, if we want to get a definition of the MDE_S_ that is equivalent to the human eye’s MDE and MPE, we have to transfer focal plane saturation thresholds to corresponding values at the position in front of the camera lens. If we additionally want to describe the extent of dazzle (size of the dazzle spot on the imaging sensor), we need a way to estimate the irradiance distribution at the focal plane quantitatively.

The calculation of the focal plane irradiance distribution for camera sensors has been already accomplished by several researchers, for example, by Schleijpen et al. [12], Benoist et al. [14] or Özbilgin et al. [29]. Their work aimed to estimate the size of a dazzle spot in cameras or thermal imagers. From the work of Schleijpen and Benoist, we can learn that it is mandatory to include scattering of light from the camera lens to explain the extent of dazzle spots at higher irradiance levels. In their earlier work, scattering was simply described by a constant referring to the irradiance level in the focal plane [12]. In later work, a scatter function of the form $a\cdot \Theta^{b}$ was introduced [14]. Using this approach, the size of the laser dazzle spot could be described very accurately. However, the calculations of dazzle spot sizes were based on the integration of the point spread function (PSF) over the area of the sensor pixels using a computer software. Özbilgin et al. used such an approach as well [29]. Unfortunately, this integration will not lead to closed-form expressions and, thus, does not suit the second constraint. Therefore, I decided to take a different path for this goal, which is described in the following sections. Furthermore, the previous work assumes that the camera lens is overfilled by the laser beam leading to a nearly homogeneous illumination of the camera lens. This is reasonable for most situations, where a dazzle laser has a large distance to the camera sensor. However, high-power laser pointers may also be used on short distances, for example to dazzle police officers at demonstrations. In such cases, the assumption of homogeneous illumination may be not fulfilled. Therefore, I will try to extent the equations also to the case, where the size of the laser beam is in the order of the size of the entrance aperture of the camera lens.

Section 2 begins with the description of the assumed dazzle scenario and the parameters that will be taken into account. In Section 3, closed-form equations to approximate the focal plane irradiance distribution are derived. This work is the prerequisite for the laser safety calculations, which are introduced in Section 4. Unfortunately, the third constraint cannot be fulfilled completely (the use of standard parameters only). Therefore, in Section 5, values for parameters are proposed that are usually not know. This includes laser-induced damage thresholds, laser saturation thresholds and scatter parameters. Section 6 presents some sample calculations.

## 2. Dazzle Scenario

**Figure 2:**
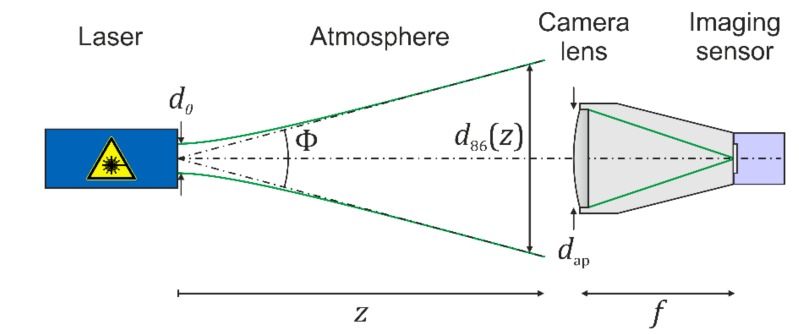
Schematic view of a dazzling scenario.

For the calculations, we assume a scenario as shown in Figure 2. A laser shall emit a beam with Gaussian beam profile and illuminates a sensor consisting of a camera lens and an imaging sensor. In Figure 2, the camera lens is depicted as a single lens, but will be treated as an optical system consisting of several optical elements.

In the further course of my investigations, I assume the laser to emit continuous-wave radiation. The laser system is described by its output power $P_{0}$ (W), wavelength λ (m), beam diameter $d_{0}$ (m) at the exit port and far-field divergence Φ (rad). One should keep in mind that there are various definitions for the laser beam diameter and divergence. In the case of Gaussian beams, most commonly the beam diameter and the divergence are related to those points of the radial profile, where the intensity has dropped to 1/e^2^ of the maximum value. For Gaussian beams, 86 percent of the laser power is encircled within this beam diameter. Therefore, the subscript “86” will be used in the further course of the manuscript, when this definition of diameter and divergence is used.

For laser safety calculations, typically the beam diameter is related to the 1/e-intensity points of the beam profile. For Gaussian beams, 63 percent of the laser power is encircled within this diameter. Beam diameter and divergence based on this definition will use the subscript “63”. The $d_{63}$-diameter is related to the $d_{86}$-diameter by

(1) $$d_{63}=\frac{d_{86}}{\sqrt{2}}$$

The advantage of using the $d_{63}$-definition is that the peak irradiance $E_{\mathrm{laser}}$ (W/m^2^) of a Gaussian beam can be calculated from the laser power $P_{\mathrm{laser}}$ (W) by dividing it by the area $A_{63}$ (m^2^) of the $d_{63}$-circle:

(2) $$E_{\mathrm{laser}}=\frac{P_{\mathrm{laser}}}{A_{63}}=\frac{P_{\mathrm{laser}}}{\frac{\pi }{4}d_{63}^{2}}=\frac{P_{\mathrm{laser}}}{\frac{\pi }{8}d_{86}^{2}}$$

The light emitted by the laser source propagates through the atmosphere to the sensors system. The atmosphere is characterized by the extinction coefficient μ (1/m), which leads to an attenuation of the laser power by a factor of

(3) $$T_{\mathrm{atm}}=\exp\left(-\mu z\right)$$

Thus, the laser power in a distance z (m) is than given by

(4) $$P_{\mathrm{laser}}\left(z\right)=P_{0}\exp\left(-\mu z\right)$$

The laser beam diameter in a distance z to the laser source can be calculated by

(5) $$d\left(z\right)=\sqrt{d_{0}^{2}+\Phi^{2}z^{2}}$$

regardless of the definition of beam diameter/divergence.

The camera lens is described by its focal length f (m), *f*-number F and aperture diameter $d_{\mathrm{ap}}$ (m), which are connected by

(6) $$F=\frac{f}{d_{\mathrm{ap}}}$$

Furthermore, to calculate the amount of stray light occurring due to the laser illumination, we use three scattering parameters, which will be described in more detail in Section 3.3. The amount of stray light also depends on the number of optical elements $N_{\mathrm{oe}}$ that form the camera lens. The optics transmittance T will also be taken into account.

The imaging sensor is characterized by several parameters: the number of pixel columns and rows $N_{\mathrm{col}}$, $N_{\mathrm{row}}$, pixel pitch p (m), fill factor ff, quantum efficiency η, full well capacity C and the integration time $t_{\exp}$ (s). We assume that the sensor system is well focused, i.e., the imaging sensor is placed at (or very near) to the focal plane of the camera lens.

## 3. Estimation of the Focal Plane Irradiance

The irradiance distribution at the focal plane of an imaging sensor illuminated by laser light is usually described by the point spread function (PSF) of the camera lens. The PSF is the Fourier transform of the optical transfer function (OTF). For example, Benoist and Schleijpen use this approach to model the size of a dazzle spot for CCD cameras [14]. They start with a diffraction limited OTF of a lens, and then they include an aberration transfer function and finally incorporate a scatter function to describe the size of a laser dazzle spot. The parameters of the scatter function are estimated by fitting the function to experimental data. With their approach, they can show a good agreement between their model and experimental data.

Here, such an approach will not be applied, since the goal is the derivation of closed-form expressions for an approximation of the focal plane irradiance. The calculation of the PSF regarding an OTF including diffraction, aberrations and scatter will certainly not lead to such closed-form expression and has to be performed using a computer.

The focal plane irradiance shall be approximated by using simple analytic expression for the diffraction irradiance. Section 3.1 describes the case of homogeneous illumination and Section 3.2 the illumination of the camera lens with a laser beam of Gaussian beam shape. Section 3.3 treats the stray light irradiance at the focal plane. I neglect aberrations, but discuss in Section 3.4 why neglecting aberrations is reasonable for laser safety calculations. Finally, in Section 3.5, the focal plane irradiance is approximated by the diffraction and the scatter component.

### 3.1. Airy Diffraction Pattern

If the laser source is located far from the camera lens, the beam diameter $d_{86}\left(z\right)$ at the position of the camera lens is much larger than the diameter of the lens aperture $d_{\mathrm{ap}}$. In this case, we can assume that the irradiance distribution at the lens aperture is homogeneous and an Airy diffraction pattern will occur in the focal plane at the position of the imaging sensor. Here, we neglect that a real lens aperture may have a polygonal shape and assume a circular shape.

The Airy diffraction pattern can be calculated by:

(7) $$E_{\mathrm{Airy}}\left(r\right)=E_{0}{\left(\frac{2J_{1}\left(x\right)}{x}\right)}^{2}\;\mathrm{with}\;x=\frac{\pi r}{\lambda F}$$

where r (m) is the radial coordinate in the focal plane and $J_{1}\left(x\right)$ is the Bessel function of first kind [30]. The peak irradiance $E_{0}$ (W) of the diffraction pattern at the focal plane is given by [30]

(8) $$E_{0}=\frac{P_{\mathrm{in}}T\pi }{4\lambda^{2}F^{2}}$$

where T is the optics transmittance and $P_{\mathrm{in}}$ (W) is the fraction of the laser power entering the lens aperture. This fraction can be calculated by

(9) $$P_{\mathrm{in}}=P_{\mathrm{laser}}\left(1-\exp\left(-\frac{2}{\nu^{2}}\right)\right)$$

where *ν* is the so-called truncation factor:

(10) $$\nu =\frac{d_{86}}{d_{\mathrm{ap}}}$$

**Figure 3:**
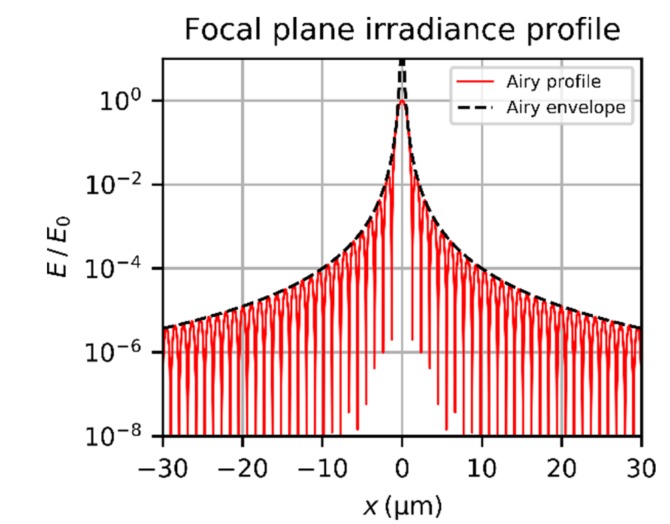
Normalized irradiance profile of an Airy diffraction pattern and its envelope according to Equation (7) and Equation (12), respectively. Calculation parameters: λ=532 nm and an *f*-number of F=2.0.

A typical irradiance profile of an Airy diffraction pattern according to Equation (7) is shown in Figure 3. The irradiance is normalized to the peak irradiance $E_{0}$. For the calculations, a laser wavelength of λ=532 nm and an F=2.0 wre used.

In order to achieve closed-form expressions, Bessel functions has to be avoided in the further equations. A simplification of Equation (7) can be derived by approximating the Bessel function by

(11) $$J_{1}\left(x\right)≅\sqrt{\frac{2}{\pi x}}\cos\left(x-\frac{3\pi }{4}\right)$$

When the oscillating cosine term is omitted, we get the envelope of the Airy diffraction pattern:

(12) $$E_{\mathrm{env}}\left(r\right)=E_{0}\cdot \frac{8\lambda^{3}F^{3}}{\pi^{4}r^{3}}=\frac{2P_{\mathrm{in}}T\lambda F}{\pi^{3}r^{3}}=\frac{2P_{\mathrm{laser}}T\lambda F}{\pi^{3}r^{3}}\left(1-\exp\left(-\frac{2}{\nu^{2}}\right)\right)$$

The envelope of the Airy diffraction pattern is also plotted in the graph of Figure 3. If the oscillating term of the Bessel function is not omitted but averaged, it is possible to calculate the mean irradiance of the diffraction rings. The average of the cosine term of Equation (11) yields only an additional factor of 0.5, which results in:

(13) $$E_{\mathrm{mean}}\left(r\right)=\frac{P_{\mathrm{in}}T\lambda F}{\pi^{3}r^{3}}=\frac{P_{\mathrm{laser}}T\lambda F}{\pi^{3}r^{3}}\left(1-\exp\left(-\frac{2}{\nu^{2}}\right)\right)$$

This simplification is reasonable to approximate the irradiance distribution at the imaging sensor due to diffraction. For both the author’s work on laser dazzle [11,19,20] and the work of other researchers [14,15,16,17,18], an oscillating Airy diffraction pattern could not be observed. The reasons for this are manifold:

1. As shown in Figure 3, the period of the oscillations of the irradiance profile is in the order of some micrometers (the radius of the first dark ring is 1.22 λF). The pixel size of most imaging sensors is typically larger than 3 µm. Thus, the camera image will show an averaged irradiance pattern.
2. As mentioned in the introduction, scattering of light at the optical elements of the camera lens has major influence on the size of the dazzle spot for high laser power. Especially in the wings of the dazzle spot, the scattered component dominates the irradiance distribution.
3. Aberrations reduce the contrast of the irradiance oscillations.
4. Laser systems may show fluctuations of laser power and have jitter in laser beam pointing, which additionally blurs the Airy diffraction pattern in the camera image.
5. In real situations, the laser system and/or the sensor system may move, for example, due to vibrations.
6. On long distances between laser and camera sensor, the atmospheric turbulence will cause an additional blur to the laser dazzle spot.

If the laser emits a Gaussian beam, the assumption of a homogeneous intensity distribution at the lens aperture is not completely fulfilled. The assumption is accepted for a truncation factor of ν>2 [31]. However, for well-collimated laser beams and short distances, one cannot rely on the Airy diffraction pattern. To extend the laser safety calculations to such situations, the diffraction of truncated Gaussian laser beams has to be included. Fortunately, there is a vast amount of literature addressing this topic [31,32].

### 3.2. Diffraction Pattern of a Truncated Gaussian Beam

In this section, I refer mainly on publications of Urey [31] and Haskal [32] treating the diffraction of truncated Gaussian beams. When comparing the equations given here to those of Urey, be aware that the equations of Urey are normalized. Furthermore, I modified all the equations used from these two publications by incorporating the optics transmittance T. Unfortunately, in Urey’s publication the symbol T stands for the truncation factor of Equation (10), whereas I use the symbol ν for this quantity in accordance with the publication of Haskal.

The focal plane irradiance of a truncated Gaussian beam is given by [32]

(14) $$E_{\mathrm{TrG}}\left(r\right)=\frac{8\pi P_{\mathrm{laser}}T}{\omega^{2}\lambda^{2}f^{2}}{\left[\int_{0}^{a}\exp\left(-\frac{\rho^{2}}{\omega^{2}}\right)J_{0}\left(\frac{2\pi }{\lambda f}\rho r\right)\rho d\rho \right]}^{2}$$

where $\omega =d_{86}/2$ (m) is the beam radius and $a=d_{ap}/2$ (m) the radius of the lens aperture. $J_{0}$ is the Bessel function of the first kind. Setting the radial coordinate r to zero in Equation (14), we can calculate the peak irradiance of the diffraction pattern of a truncated Gaussian beam (see Equation (6) of [31] or Equation (11) of [32]):

(15) $$E_{0}\left(\nu \right)=\frac{P_{\mathrm{in}}T\pi }{4\lambda^{2}F^{2}}\cdot \frac{2\nu^{2}{\left[1-\exp\left(-\frac{1}{\nu^{2}}\right)\right]}^{2}}{1-\exp\left(-\frac{2}{\nu^{2}}\right)}=\frac{P_{\mathrm{laser}}T\pi }{4\lambda^{2}F^{2}}\cdot 2\nu^{2}{\left[1-\exp\left(-\frac{1}{\nu^{2}}\right)\right]}^{2}$$

This equation is similar to Equation (8) for the peak irradiance of the Airy diffraction pattern; the equation for the peak irradiance of a truncated Gaussian beam is modified by a term depending only on the truncation factor ν. Equivalent to the Airy diffraction pattern, the irradiance distribution of the diffraction rings can be approximated by the envelope using the same simplification. For truncated Gaussian beams, the envelope of the diffraction ring irradiance is given by:

(16) $$E_{\mathrm{env}}\left(r\right)=\frac{2P_{\mathrm{in}}T\lambda F}{\pi^{3}r^{3}}\cdot \frac{\frac{2}{\nu^{2}}\exp\left(-\frac{2}{\nu^{2}}\right)}{1-\exp\left(-\frac{2}{\nu^{2}}\right)}=\frac{2P_{\mathrm{laser}}T\lambda F}{\pi^{3}r^{3}}\cdot \frac{2}{\nu^{2}}\exp\left(-\frac{2}{\nu^{2}}\right)$$

Again, the result is similar to the result for the Airy diffraction pattern given by Equation (12) and differs by a term depending only on the truncation factor ν. Introducing again a factor of 0.5, we get the mean irradiance of the diffraction rings $E_{\mathrm{mean}}\left(r\right)=0.5\cdot E_{\mathrm{env}}\left(r\right)$ (see also Equation (15) of reference [31]):

(17a) $$E_{\mathrm{mean}}\left(r\right)=\frac{P_{\mathrm{in}}T\lambda F}{\pi^{3}r^{3}}\cdot \frac{\frac{2}{\nu^{2}}\exp\left(-\frac{2}{\nu^{2}}\right)}{1-\exp\left(-\frac{2}{\nu^{2}}\right)}=\frac{P_{\mathrm{laser}}T\lambda F}{\pi^{3}r^{3}}\cdot \frac{2}{\nu^{2}}\exp\left(-\frac{2}{\nu^{2}}\right)$$

or, expressed in diffraction or viewing angle by using r=fθ,

(17b) $$E_{\mathrm{mean}}\left(\Theta \right)=\frac{P_{\mathrm{in}}T\lambda F}{\pi^{3}f^{3}}\cdot \frac{1}{\Theta^{3}}\cdot \frac{\frac{2}{\nu^{2}}\exp\left(-\frac{2}{\nu^{2}}\right)}{1-\exp\left(-\frac{2}{\nu^{2}}\right)}=\frac{P_{\mathrm{laser}}T\lambda F}{\pi^{3}f^{3}}\cdot \frac{1}{\Theta^{3}}\cdot \frac{2}{\nu^{2}}\exp\left(-\frac{2}{\nu^{2}}\right)$$

According to Urey, this approximation works well beyond the second zero crossing of the diffraction pattern [31].

**Figure 4:**
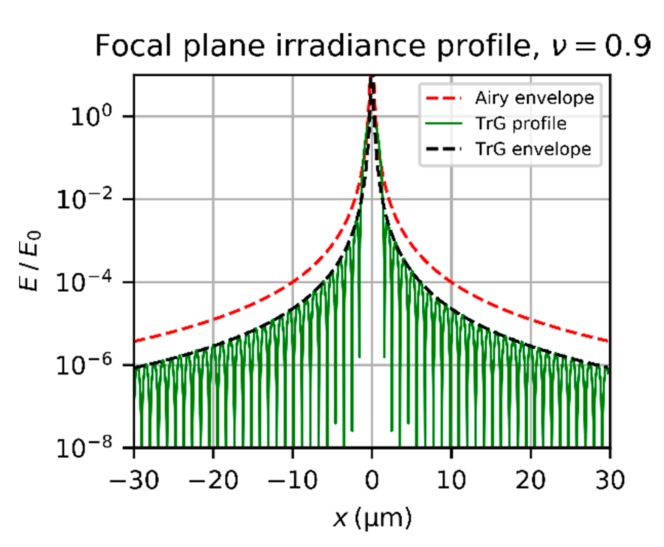
Irradiance profile of the diffraction pattern of a truncated Gaussian beam (TrG) and its envelope according to Equation (14) and Equation (16), respectively. Calculation parameters: λ=532 nm, f=50 mm, F=2.0, ν=0.9. Additionally, the envelope of the Airy diffraction pattern is plotted according to Equation (12) for the same incident power $P_{\mathrm{in}}$. The curves are normalized using the peak irradiance $E_{0}$ of an Airy diffraction pattern for same incident power $P_{\mathrm{in}}$.

Figure 4 shows a plot of the irradiance profile of a truncated Gaussian beam and its envelope according to Equation (14) and Equation (16), respectively. Additionally, the envelope for the Airy diffraction pattern according to Equation (12) is plotted assuming the same incident power $P_{\mathrm{in}}$. The envelope of the truncated Gaussian beam for ν=0.9 is a factor 4.8 below the envelope of the Airy diffraction pattern.

**Figure 5:**
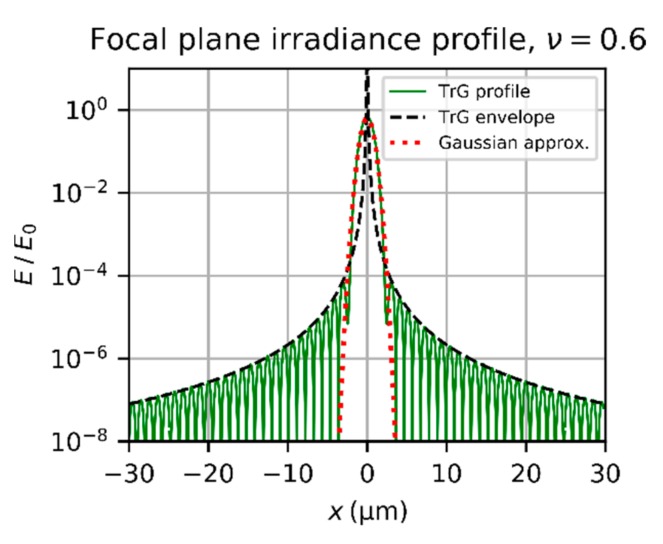
Irradiance profile of the diffraction pattern of a truncated Gaussian beam (TrG) and its envelope according to Equation (14) and Equation (16), respectively. Calculation parameters: λ=532 nm, f=50 mm, F=2.0, ν=0.6. Additionally, a Gaussian approximation for the central lobe according to Equation (18) is plotted. The curves are normalized using the peak irradiance $E_{0}$ of an Airy diffraction pattern for same incident power $P_{\mathrm{in}}$.

When the truncation factor decreases, the central lobe of the diffraction pattern gets wider. This is shown in Figure 5, where the diffraction pattern of a truncated Gaussian beam is plotted for a truncation factor ν=0.6. We can also see from this graph that the envelope does not fit to the central lobe properly.

The central lobe of the diffraction pattern can be approximated by a Gaussian curve:

(18a) $$E_{\mathrm{GA}}\left(r\right)=E_{0}\left(\nu \right)\exp\left(-8\frac{r^{2}}{d_{\mathrm{spot}}^{2}}\right)$$

or, expressed in diffraction or viewing angle,

(18b) $$E_{\mathrm{GA}}\left(\Theta \right)=E_{0}\left(\nu \right)\exp\left(-8\frac{{\left(f\Theta \right)}^{2}}{d_{\mathrm{spot}}^{2}}\right)$$

where $d_{\mathrm{spot}}$ is given by [31]

(19) $$d_{\mathrm{spot}}=K\lambda F$$

The factor *K* depends on the truncation factor ν [31]:

(20) $$K=\frac{0.97}{\nu }\sqrt{\frac{\exp\left(1\right)}{1-\exp\left(-\frac{1}{\nu^{2}}\right)}-1}$$

or

(21) $$K=\left\{\begin{array}{cc} & \frac{1.27}{\nu },\;\nu <0.5\\ 1.654-\frac{0.105}{\nu }+ & \frac{0.28}{\nu^{2}\;},\;\;\nu >0.4 \end{array}\right.$$

In Figure 5, the Gaussian approximation of the central lobe according to Equation (18) is plotted as red dotted line. Finally, the focal plane irradiance due to diffraction of a truncated Gaussian beam could be approximated by using the Gaussian approximation $E_{\mathrm{GA}}\left(r\right)$ for the central lobe and the mean irradiance of the diffraction rings $E_{\mathrm{mean}}\left(r\right)$ for the wings. For this, one has to calculate the point of intersection $\Theta_{\mathrm{pi}}$ of both equations by solving $E_{\mathrm{GA}}\left(r_{\mathrm{pi}}\right)=E_{\mathrm{mean}}\left(r_{\mathrm{pi}}\right)$, which leads to:

(22) $$r_{\mathrm{pi}}{}^{3}\cdot \exp\left(-8\frac{r_{\mathrm{pi}}{}^{2}}{d_{\mathrm{spot}}^{2}}\right)=\frac{4\lambda^{3}F^{3}}{\pi^{4}}\cdot \frac{\exp\left(-\frac{2}{\nu^{2}}\right)}{\nu^{4}{\left[1-\exp\left(-\frac{1}{\nu^{2}}\right)\right]}^{2}}$$

The diffraction irradiance can then be approximated by:

(23) $$E_{d}\left(r\right)=\left\{\begin{array}{c} E_{\mathrm{GA}}\left(r\right),\;\;\left|r\right|\leq r_{\mathrm{pi}}\\ E_{\mathrm{mean}}\left(r\right),\;\;\left|r\right|>r_{\mathrm{pi}} \end{array}\right.$$

**Figure 6:**
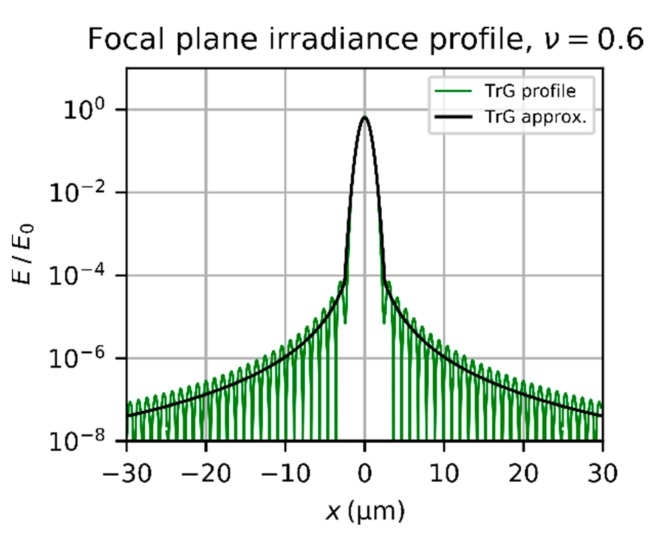
Irradiance profile of the diffraction pattern of a truncated Gaussian beam (TrG) and its approximation according to Equation (14) and Equation (23), respectively. Calculation parameters: λ=532 nm, f=50 mm, F=2.0, ν=0.6. The curves are normalized using the peak irradiance $E_{0}$ of an Airy diffraction pattern for same incident power $P_{\mathrm{in}}$.

Figure 6 shows a plot of $E_{d}$ according to Equation (23).

Unfortunately, there is no analytical solution for Equation (22) and $r_{\mathrm{pi}}$ can only be approximated numerically. Table 1 lists some values of the point of intersection $r_{\mathrm{pi}}=f\Theta_{\mathrm{pi}}$ for various values of wavelength, *f*-number and truncation factor. The wavelengths were chosen to match those of common laser pointers. We can see that the point of intersection increases with increasing wavelength, increasing *f*-number and decreasing truncation factor. The largest value in Table 1 is ~54 µm, which would correspond to 9–15 sensor pixels for typical values of the pixel pitch (3.5–6 µm). Certainly, larger values can occur for extreme situations, for example, when a laser beam with small diameter enters a camera lens with large aperture. For λ=660 nm, F=11 and ν=0.1, I calculated a value $r_{\mathrm{pi}}\approx 470\;µm$, which would corresponds to roughly 100 sensor pixels. Such a situation could occur for very short distances of the laser source to the sensor system. For comparison, Table 1 lists also the spot size radius $d_{\mathrm{spot}}/2$ according to Equation (19).

Since the point of intersection $r_{\mathrm{pi}}$ cannot be calculated analytically, we can simply take the maximum value of $E_{\mathrm{mean}}\left(\Theta \right)$ and $E_{\mathrm{GA}}\left(\Theta \right)$ to approximate the irradiance distribution. However, we have to take into account that $E_{\mathrm{mean}}\left(\Theta \right)$ has a singularity at zero. Thus, the irradiances values has to be limited to $E_{0}\left(\nu \right)$, which leads to the following approximation of the diffraction irradiance:

(24) $$E_{d}\left(\Theta \right)=\max\left[E_{\mathrm{GA}}\left(\Theta \right),\;\min(E_{\mathrm{mean}}\left(\Theta \right),\;E_{0}\left(\nu \right))\right]$$

### 3.3. Stray Light Irradiance

Beyond the diffraction of laser light at the camera lens’ aperture, the scattering of light at the optical elements and the housing of the camera lens has a major influence on the distribution of light on the imaging sensor. Stray light in optical systems is typically modelled with help of optical design software, like for example ZEMAX, ASAP, or FRED. However, for the laser safety calculations, we want to describe the scattering of light in the camera lens also by closed-form expressions.

In the past, a lot of work was done to investigate light scattering caused by the surface roughness of optical elements [33,34,35,36]. Of course, it cannot be expected that the complex process of light scattering at the optical elements and the housing, including multiple reflections, can be described completely by simple equations. However, if the calculations are limited to scattering at the rough surfaces of optical elements and neglect multiple reflections between the optical elements of the camera lens and light scattering at the housing, it is possible to come to an analytical description of the stray light. Such calculations were done by Peterson, who set up analytical equations for the stray light irradiance at the focal plane of an optical system [37]. I follow Peterson’s work and make further simplifications to his approach to reach simple closed-form expressions for the distribution of scattered light in the focal plane of an optical system.

Light scattering at surfaces is described by the bidirectional scatter distribution function (BSDF). The BSDF is the ratio of the scattered radiance (W/(sr m^2^)) to the incident irradiance (W/m^2^) of a scattering surface. According to Peterson, light scattering at the rough surface of optical elements is described by a Harvey scatter model. The BSDF of that model is based in the two parameters b and s and is given by the following equation [37]

(25) $$\rho =b{\left(100\cdot \left|\sin\left(\Theta \right)-\sin\left(\Theta_{0}\right)\right|\right)}^{s}$$

In Equation (25), $\sin\left(\Theta \right)-\sin\left(\Theta_{0}\right)$ describes the scatter angle related to the incident light or, in case of mirrors, related to the specularly reflected light. The scatter parameter s defines the slope of the BSDF in a logarithmic plot and the parameter b (sr^-1^) the value of the BSDF for a scatter angle $\left|\sin\left(\Theta \right)-\sin\left(\Theta_{0}\right)\right|=0.01$. The parameters are typically in the range $0.01\;{\mathrm{sr}}^{-1}\leq b\leq 1\;{\mathrm{sr}}^{-1}$ and −3≤s≤−1. As a side note, the empirical approach $a\cdot \Theta^{b}$ used by Benoist et al. [14] (mentioned in the introduction) corresponds to Equation (25), when the small angle approximation $\sin\left(\Theta \right)\approx \Theta$ and an incidence angle $\Theta_{0}=0$ is used.

This kind of BSDF has a singularity for $\sin\left(\Theta \right)-\sin\left(\Theta_{0}\right)=0$. To avoid this, a third scatter parameter l can be introduced, which results in a 3-parameter Harvey scatter model [37]. The BSDF for such a modified BSDF is given by equation

(26) $$\rho =b_{0}{\left[1+{\left(\frac{\sin\left(\Theta \right)-\sin\left(\Theta_{0}\right)}{l}\right)}^{2}\right]}^{\frac{s}{2}}$$

The so-called *shoulder parameter* l (rad) defines the scatter angle at which the BSDF changes from the decaying region with slope s to a constant region with value $b_{0}$. Typical values of l may be in the range of $10^{-4}\;\mathrm{rad}\leq l\leq 0.01\;\mathrm{rad}$ [38]. The parameter $b_{0}$ is linked to the parameters b and s of the 2-parameter scatter model by

(27) $$b_{0}=b\;{\left(100\cdot l\right)}^{s}$$

Based on the last two equations, Peterson estimates the scattered irradiance in the focal plane of an optical system [37]. The optical system shall consist of a number of scattering elements (e.g., lenses). According to Peterson, the scatter irradiance of the *j*^th^ scattering element using the 3-parameter scatter model is expressed by the equation

(28) $$E_{s,j}\left(r\right)=\pi T\;NA^{2}\frac{a_{\mathrm{ent}}^{2}}{a_{j}^{2}}b_{0}{\left[1+{\left(\frac{NA\;r}{la_{j}}\right)}^{2}\right]}^{s/2}E_{\mathrm{ent}}$$

where NA is the numerical aperture, $a_{\mathrm{ent}}$ (m) the radius of the beam at the first scattering element and $a_{j}$ (m) the radius of the beam at the *j*^th^ scattering element. $E_{\mathrm{ent}}$ (W/m^2^) is the incident irradiance. The total stray light irradiance can be calculated by adding up the contributions of the single scattering elements:

(29) $$E_{s}\left(r\right)=\underset{j}{\sum }E_{s,j}\left(r\right)$$

Using Equation (29), the stray light in the focal plane of a camera lens comprising a number of optical elements $N_{\mathrm{oe}}$ is described. Each optical element of the camera lens may have two scattering surfaces (e.g., lenses) or one scattering surface (e.g., mirrors). Usually, for commercial off-the-shelf camera lenses, the manufacturer does not reveal the exact optical design. Thus, the exact number of scattering surfaces cannot be stated without inside knowledge. For example, if two lenses are glued together to form an achromatic doublet lens, they have three scattering surfaces, whereas an air-spaced doublet has four scattering surfaces. Thus, I make a first approximation.

**Approximation 1.** All optical elements of the camera lens are lenses with two scattering surfaces. The number of scattering surfaces is then given by:

(30) $$N_{\mathrm{ss}}=2\cdot N_{\mathrm{oe}}$$

If knowledge about the real number of scattering surfaces is available, of course this number can be used instead of applying Equation (30).

Without detailed information about the optical design of the camera lens, it is usually not known exactly, how the beam size $a_{j}$ varies at the different scattering surfaces. Thus, I have to introduce a second approximation.

**Approximation 2.** The beam radius is assumed to be the same at all optical elements and equal to the entering beam size:

(31) $$a_{j}=a_{\mathrm{ent}}=\mathrm{const}$$

In Appendix A, I describe why this approximation is reasonable. The radius of the entering beam $a_{\mathrm{ent}}$ depends on the distance of the laser source to the sensor system. For short distances, where the laser beam diameter is smaller than the diameter of the lens aperture, $a_{\mathrm{ent}}$ is set to the effective beam radius $d_{63}/2$, whereas for larger distances, $a_{\mathrm{ent}}$ is limited to the radius of the lens aperture $d_{\mathrm{ap}}/2$:

(32a) $$a_{j}=\left\{\begin{array}{c} \frac{d_{63}}{2},\;\;d_{63}<d_{\mathrm{ap}}\\ \frac{d_{\mathrm{ap}}}{2},\;\;d_{63}\geq d_{\mathrm{ap}} \end{array}\right.$$

Using $d_{\mathrm{ap}}=\frac{f}{F}$ and $\nu =\frac{d_{86}}{d_{\mathrm{ap}}}=\frac{d_{86}F}{f}=\frac{\sqrt{2}d_{63}F}{f}$, we can rewrite Equation (32a) as:

(32b) $$a_{j}=\left\{\begin{array}{c} \frac{\nu }{\sqrt{2}}\cdot \frac{f}{2F},\;\;\nu <\sqrt{2}\\ \frac{f}{2F},\;\;\nu \geq \sqrt{2} \end{array}\right.=\nu^{*}\cdot \frac{f}{2F}$$

The parameter $\nu^{*}$ is defined as

(33) $$\nu^{*}=\min\left(1,\frac{\nu }{\sqrt{2}}\right)$$

For the entering irradiance $E_{\mathrm{ent}}$ in Equation (28), we then use the mean value:

(34) $$E_{\mathrm{ent}}=\bar{E_{\mathrm{ent}}}=\frac{P_{\mathrm{in}}}{\pi a_{j}^{2}}=\frac{4P_{\mathrm{in}}F^{2}}{\pi f^{2}}\cdot \frac{1}{{\left(v^{*}\right)}^{2}}$$

Using both approximations, Equation (32b) and (34) and substituting the numerical aperture by $NA=1/\left(2F\right)$, the scattering irradiance of Equation (29) simplifies to:

(35a) $$E_{s}\left(r\right)=\frac{P_{\mathrm{in}}TN_{\mathrm{ss}}b_{0}}{f^{2}}\frac{1}{{\left(v^{*}\right)}^{2}}{\left[1+\frac{1}{{\left(v^{*}\right)}^{2}}\cdot {\left(\frac{r}{lf}\right)}^{2}\right]}^{\frac{s}{2}}\;=\frac{P_{\mathrm{laser}}TN_{\mathrm{ss}}b_{0}}{f^{2}}\frac{1}{{\left(v^{*}\right)}^{2}}{\left[1+\frac{1}{{\left(v^{*}\right)}^{2}}{\left(\frac{r}{lf}\right)}^{2}\right]}^{\frac{s}{2}}\cdot \left(1-\exp\left(-\frac{2}{\nu^{2}}\right)\right)$$

or, expressed in scatter or viewing angle,

(35b) $$E_{s}\left(\Theta \right)=\frac{P_{\mathrm{in}}TN_{\mathrm{ss}}b_{0}}{f^{2}}\frac{1}{{\left(v^{*}\right)}^{2}}{\left[1+\frac{1}{{\left(v^{*}\right)}^{2}}{\left(\frac{\Theta }{l}\right)}^{2}\right]}^{\frac{s}{2}}\;=\frac{P_{\mathrm{laser}}TN_{\mathrm{ss}}b_{0}}{f^{2}}\frac{1}{{\left(v^{*}\right)}^{2}}{\left[1+\frac{1}{{\left(v^{*}\right)}^{2}}{\left(\frac{\Theta }{l}\right)}^{2}\right]}^{\frac{s}{2}}\cdot \left(1-\exp\left(-\frac{2}{\nu^{2}}\right)\right)$$

**Figure 7:**
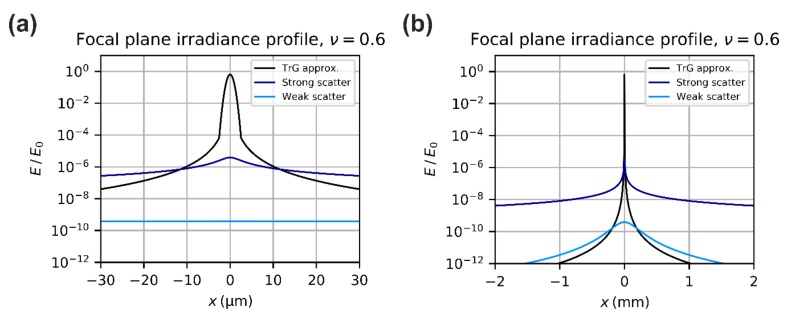
Approximation of the irradiance profile of a truncated Gaussian beam diffraction pattern according to Equation (23) and irradiance profile of the scattered radiation according to Equation (35). Calculation parameters: λ=532 nm, f=50 mm, F=2.0, ν=0.6,$\;N_{ss}=12$. “Strong scatter”: s=−1, b=1, $l=10^{-4}$, “Weak scatter”: s=−3, b=0.01, l=0.01. The curves are normalized using the peak irradiance $E_{0}$ of an Airy diffraction pattern for same incident power $P_{\mathrm{in}}$. (**a**) x-coordinate ranges from −30 µm to +30 µm. (**b**) x-coordinate ranges from −2 mm to +2 mm.

In Figure 7, two plots show the irradiance profile for diffraction, approximated according Equation (23), and for scattering, approximated by Equation (35). In the left graph, the x-coordinates range from −30 µm to +30 µm as in the graphs above. In the right graph, the x-coordinates are expanded to a range from −2 mm to +2 mm, what matches roughly the longer edge of a ¼-inch imaging sensor. The scatter curves represent two sets of scattering parameters and correspond to two extreme cases (strong scatter and weak scatter).

The scatter parameters are wavelength dependent. In a technical note, which treats scattering in the optical design software ASAP [39], the wavelength dependency is described by the equations:

(36) $$b\left(\lambda \right)=b\left(\lambda_{0}\right){\left(\frac{\lambda_{0}}{\lambda }\right)}^{4+s}\;s\left(\lambda \right)=s\left(\lambda_{0}\right)\;l\left(\lambda \right)=l\left(\lambda_{0}\right)\left(\frac{\lambda }{\lambda_{0}}\right)$$

Reference [39] states that these wavelength scaling laws are valid only “over a limited range of λ”, but limits are not given. Wein, for example, states that the “wavelength scaling approximately predicts the scattering in the visible but not in the far-infrared” [40].

The scatter parameters b or $b_{0}$, s and l are usually not know for the optical elements of camera lenses; they have to be estimated experimentally. In principle, measurements of the scatter parameters have to be done for each single type of camera lens. For the laser safety calculations, however, the aim is to derive a set of the scatter parameters b, s and l that can be applied for common camera lenses. This is treated in Section 5.1, where scatter parameters for different camera lenses are presented.

Generally, all the equations noted here for describing scattering are based on the assumption of surface scatter from clean, smooth surfaces. The equations do not describe multiple scattering or scattering at the housing of the camera lens. Greynolds calculated the amount of scattering power impinging on the detector of a mirror telescope resulting from various scattering effects [41]. For small off-axis angles (< 20°), the main contribution of power at the detector is from scattering at the primary mirror. The stray light reaching the detector caused by scattering at the tube and subsequently by the primary mirror is several orders of magnitude lower. Therefore, I expect that the formulas presented here give a reasonable approximation for light scattering in camera lenses.

### 3.4. Aberrations

Aberrations in camera lenses usually lead to a larger focal spot size and to a decreased peak irradiance of the spot. However, I will neglect aberrations for the laser safety calculations. There are various rationales, why this is reasonable but also some practical reasons why I want to proceed in this way:

1. One goal of the laser safety calculations is to estimate the onset of laser damage. By neglecting the aberrations, a higher peak irradiance is estimated, which leads to a lower value of the calculated MPE_S_. This can be interpreted as a safety factor, equivalent to the MPE for the human eye. The MPE for the human eye is derived from experimentally estimated ED_50_-values for eye damage, and is defined as a value that is usually a factor of 10 below these ED_50_ threshold values.
2. Regarding laser dazzle of sensors, the other aim of the laser safety calculation is to estimate the dazzle spot size. In this case, the aberrations have a minor influence, since the size of the dazzle spot at larger dazzle levels is mainly caused by the stray light. Only for very low laser powers, slightly above the onset of laser dazzle, this assumption will cause some error in the dazzle spot size by neglecting aberrations.
3. For commercial camera lenses, information on aberrations is typically provided, if at all, in form of diagrams. To transfer this information to values is complex.
4. The treatment of aberrations using analytical expressions would increase the complexity of the equations for laser safety calculations a lot.

Therefore, in order to keep calculations simple, aberrations will be neglected for the laser safety calculations. If one wants to include aberrations to a certain degree, a possibility would be to use the Strehl ratio $D_{S}$, which is the ratio of real peak irradiance to the peak irradiance of an aberration free system. This would mean that we extend Equation (15) for the peak irradiance of the truncated Gaussian diffraction pattern by the Strehl ratio: $E_{0}^{*}\left(\nu \right)=D_{S}\;E_{0}\left(\nu \right)$. However, for camera lenses with good optical quality, a Strehl ratio of $D_{S}>0.8$ can be assumed. This means that the calculated values of MPE_S_ may be a factor of 1.25 too high, which is acceptable as a safety factor.

### 3.5. Total Focal Plane Irradiance

Using the results of the preceding sections, the focal plane irradiance distribution can be approximated by the diffracted irradiance $E_{d}$ and the scattered irradiance $E_{s}$. We can take into account that the scattering process transfers power from the diffracted light to scattered light using the so-called total integrated scatter (TIS). The value TIS is usually defined as the ratio of diffuse reflectance to total reflectance (specular + diffuse) [42]. In the case of refracting optical elements (lenses), TIS corresponds to the ratio of scattered power to total transmitted power:

(37) $$TIS=\frac{\mathrm{scattered}\;\mathrm{power}}{\mathrm{total}\;\mathrm{transmitted}\;\mathrm{power}}=\frac{P_{s}}{TP_{\mathrm{in}}}$$

For the 3-parameter Harvey scatter model, the total integrated scatter can be calculated by [39]:

(38) $$TIS=\left\{\begin{array}{c} 2\pi b\frac{100^{s}}{s+2}\;\left[{\left(1+l^{2}\right)}^{\frac{s+2}{2}}-{\left(l^{2}\right)}^{\frac{s+2}{2}}\right],\;\;s\neq -2\\ 2\pi b\frac{{\left(100l\right)}^{s}}{2}l^{2}\ln\left(1+\frac{1}{l^{2}}\right),\;\;\;s=-2 \end{array}\right.$$

Using extreme values for the scatter parameters of $b=1\;{\mathrm{sr}}^{-1}$, s=−1 and $l=10^{-4}\;\mathrm{rad}$, the maximum value of the total integrated scatter, according to Equation (38), is $TIS_{\max}\approx 6.3\%$ for a single scattering surface.

The fraction of diffracted power is given by $\eta_{d}=1-TIS$ for a single scattering surface. For a camera lens with $N_{\mathrm{ss}}$ scattering surfaces, the fraction of diffracted power $\eta_{d}$ is then

(39) $$\eta_{d}={\left(1-TIS\right)}^{N_{\mathrm{ss}}}$$

The focal plane irradiance distribution can be calculated by the sum of diffraction irradiance and the stray light irradiance:

(40) $$E_{\mathrm{fp}}\left(\Theta \right)=\eta_{d}E_{d}\left(\Theta \right)+E_{s}\left(\Theta \right)$$

**Figure 8:**
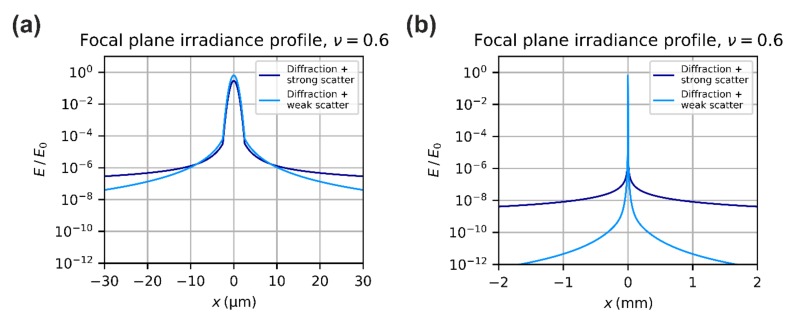
Approximation of the focal plane irradiance profile according to Equation (40). Calculation parameters: λ=532 nm, f=50 mm, F=2.0, ν=0.6, $N_{\mathrm{ss}}=12$. “Strong scatter”: s=−1, b=1, $l=10^{-4}$, “Weak scatter”: s=−3, b=0.01, l=0.01. The curves are normalized using the peak irradiance $E_{0}$ of an Airy diffraction pattern for same incident power $P_{\mathrm{in}}$. (**a**) x-coordinate ranges from −30 µm to +30 µm. (**b**) x-coordinate ranges from −2 mm to +2 µm.

Figure 8 presents two plots that show the irradiance profile according to Equation (40). Again, in the left graph, the x-coordinates range from −30 µm to +30 µm and in the right graph, the x-coordinates are expanded to a range from −2 mm to +2 mm. The two curves shown correspond to two sets of scattering parameters.

## 4. Laser safety Calculations for Sensors

**Figure 9:**
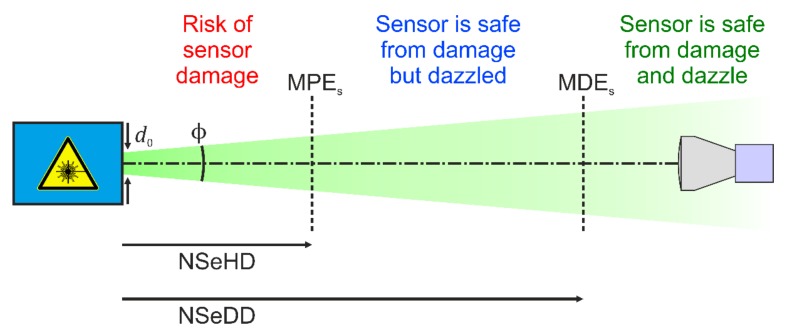
Hazard distances for imaging sensors: Nominal Sensor hazard Distance (NSeHD) and Nominal Sensor Dazzle Distance (NSeDD).

Section 3 laid the basis for the laser safety calculations. In Section 4.1, I start with the derivation of a Maximum Permissible Exposure for a Sensor (MPE_S_) and continue in Section 4.2 with the definition of Maximum Dazzle Exposure for a Sensor (MDE_S_). Section 4.3 treats the hazard distances corresponding to the MPE_S_ and the MDE_S_. I define the hazard distances for imaging sensors in the same way as it is defined for the human eye. The Nominal Sensor Hazard Distance (NSeHD) is the distance of the sensor system to the laser source, where the incident peak irradiance is equal to the MPE_S_. The Nominal Sensor Dazzle Distance (NSeDD) is the distance of the sensor system to the laser source, where the incident peak irradiance is equal to the MDE_S_. The hazard distances are illustrated in Figure 9. In Section 4.4, I calculate the size of a laser dazzle spot in a camera image.

The equations derived in Section 3 are closed-form expressions to approximate the irradiance distribution in the focal plane of a camera lens. However, their use in the stated form would lead to complex laser safety calculations. Therefore, I will make some simplifications to reduce the complexity.

To calculate, for example, the onset of laser damage or dazzle, the peak irradiance in the focal plane has to be known, which is given by $E_{\mathrm{fp}}\left(0\right)=\eta_{d}E_{0}\left(\nu \right)+E_{s}\left(0\right)$, see Equation (40). Looking at Figure 7, we can expect that the scatter contribution to the peak irradiance is several orders of magnitude lower than the contribution of the diffracted radiation. This leads us to a first simplification.

**Simplification S1.** The scatter contribution $E_{s}\left(0\right)$ is neglected for laser safety calculations that are based on the focal plane peak irradiance.

For laser safety calculations that depend on the spatial distribution of light (e.g., the estimation of dazzle spot size), scattering of light will not be neglected.

For the same reason as discussed in Section 3.4, treating aberrations, I introduce a second simplification.

**Simplification S2.** The fraction of diffracted power $\eta_{d}$ is neglected and its value is set to 1.

I treat simplification S2 as a safety factor equivalent to laser safety calculations for the human eye (relationship between ED_50_ and MPE). Such a simplification is also used in the work of Schleijpen et al. [12]. While simplification S1 will reduce the estimated peak irradiance, simplification S2 will increase it. For typical camera lenses, the increase by simplification S2 should overcompensate the decrease of simplification S1 (see Appendix B.1 for more details).

A third simplification is applied for the spatial distribution of the focal plane irradiance.

**Simplification S3.** For laser safety calculations based on the spatial distribution of light, the Gaussian approximation of the central lobe is neglected. The diffraction irradiance is described by the mean irradiance of the diffraction rings only: $E_{d}\left(\Theta \right)\approx E_{\mathrm{mean}}\left(\Theta \right)$.

Background of simplification S3 is that the spatial irradiance profile will be used later to estimate the MDE_S_ and the size of a dazzle spot. These values are usually only of interest, when the dazzle spot fills a considerable amount of the sensor’s field of view, i.e., the radius of the dazzle spot is greater than several tens of pixels. Very small dazzle spot sizes in the order of only some pixels are usually not an issue for a sensor user.

In the following, the equations for MPE_S_ and MDE_S_ will be derived. Due to the simplifications made here, these equations will only be valid within certain limits. In Appendix B, I examine the limits of applicability in more detail.

### 4.1. Maximum Permissible Exposure for a Sensor

Equivalent to laser safety calculations for the human eye, I am only interested in the onset of sensor damage. I do not treat the morphology of laser damage or the different effects that can occur for laser power far above the damage threshold like the failure of complete pixel rows or columns. I define the Maximum Permissible Exposure for a Sensor (MPE_S_) as the incident irradiance at the camera lens that can lead to a (minimal) damage of the imaging sensor.

The maximum incident laser power to prevent sensor damage can be found by equalizing the focal plane peak irradiance $E_{\mathrm{fp}}$ given by Equation (40) and the laser induced damage threshold $E_{\mathrm{dam}}$. By applying simplifications S1 and S2 mentioned above this leads to

(41) $$E_{\mathrm{fp}}\left(0\right)\approx E_{0}\left(\nu \right)=E_{\mathrm{dam}}$$

Performing some transformations results in

(42) $$P_{\mathrm{laser},\max}=E_{\mathrm{dam}}\cdot \frac{2\lambda^{2}F^{2}}{T\pi }\frac{\frac{1}{\nu^{2}}}{{\left[1-\exp\left(-\frac{1}{\nu^{2}}\right)\right]}^{2}}$$

The maximum value of the allowed incident irradiance is then

(43) $$MPE_{S}=\frac{8P_{\mathrm{laser},\max}}{\pi d_{86}^{2}}=E_{\mathrm{dam}}\cdot \frac{16\lambda^{2}F^{2}}{T\pi^{2}d_{86}^{2}}\frac{\frac{1}{\nu^{2}}}{{\left[1-\exp\left(-\frac{1}{\nu^{2}}\right)\right]}^{2}}$$

Using

(44) $$\nu =\frac{d_{86}}{d_{lens}}=\frac{d_{86}F}{f}\Leftrightarrow d_{86}=\frac{\nu f}{F}$$

we finally get

(45a) $$MPE_{S}=E_{\mathrm{dam}}\cdot \frac{16\lambda^{2}F^{4}}{T\pi^{2}f^{2}}{\left[\frac{\frac{1}{\nu^{2}}}{1-\exp\left(-\frac{1}{\nu^{2}}\right)}\right]}^{2}$$

Looking at Equation (45a), we can see that the MPE_S_ depends on the truncation factor, which means that this quantity thus depends on the distance of the laser to the sensor. The lowest value of the MPE_S_ (worst case) occurs for ν→∞:

(45b) $$MPE_{S,\min}=\underset{\nu \to \infty }{\lim}MPE_{S}=E_{\mathrm{dam}}\cdot \frac{16\lambda^{2}F^{4}}{T\pi^{2}f^{2}}$$

The minimum value of the MPE_S_ according Equation (45b) should be used for laser safety calculations regarding scenarios, where the distance of the laser source to the sensor is not known (e.g., outdoor use of camera systems). For a specific scenario (e.g., the use of a surveillance camera inside a room), one could estimate the maximum value of the truncation factor ν that may occur and use Equation (45a) to calculate a less conservative estimate of the MPE_S_.

In order to apply Equation (45a) and Equation (45b), the laser induced damage threshold (LIDT) of the imaging sensor has to be known. Typical LIDT values of CCD and CMOS cameras for continuous-wave laser irradiation are stated in Section 5.2.

### 4.2. Maximum Dazzle Exposure for a Sensor

For the human eye, the maximum dazzle exposure is not just a single value but is specified for different dazzle levels [26]. The dazzle levels range from very low to low, medium, and high, which corresponds to angular dazzle fields of 2°, 10°, 20°, and 40°, respectively. For imaging sensors, an equivalent definition of such fixed values for the dazzle field is not useful since the sensor’s field of view (FOV) changes with the focal length of the camera lens.

**Figure 10:**
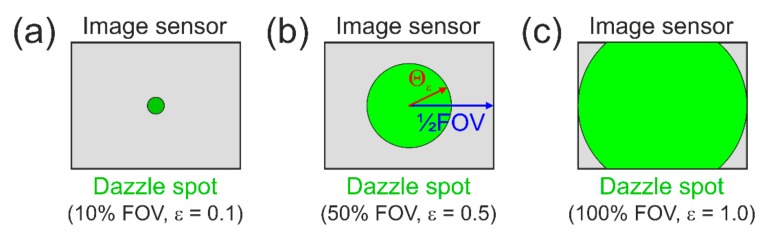
Definition of the dazzle level as fraction of the sensor’s field of view that is dazzled. (**a**) Dazzle level ϵ=0.1, (**b**) dazzle level ϵ=0.5, (**c**) dazzle level ϵ=1.0.

Thus, I propose to define the Maximum Dazzle Exposure for a Sensor (MDE_S_) in terms of the fraction ϵ of the sensor’s FOV that is dazzled. This means, e.g., that for an incident irradiance of $MDE_{S}\left(\epsilon =0.1\right)$, $MDE_{S}\left(\epsilon =0.5\right)$ and $MDE_{S}\left(\epsilon =1.0\right)$, a tenth of the FOV, half of the FOV and the full FOV is dazzled, respectively. Figure 10 illustrates that approach. The fraction ϵ shall be understood as the diameter of the dazzle spot divided by the size of the longer side of the imaging sensor.

Using this definition, the angular radius of the dazzle spot would be

(46) $$\Theta_{\epsilon }=\epsilon \cdot \frac{FOV}{2}$$

where the field of view of the camera sensor could be calculated by

(47) $$FOV=\frac{\max\left(N_{\mathrm{col}},\;N_{\mathrm{row}}\right)\cdot p}{f}$$

The value $MDE_{s}$ can be derived by equating the focal plane irradiance $E_{\mathrm{fp}}\left(\Theta_{\epsilon }\right)$ of Equation (40) at the angle $\Theta_{\epsilon }$ to the saturation irradiance $E_{\mathrm{sat}}$:

(48) $$E_{\mathrm{fp}}\left(\Theta_{\epsilon }=\epsilon \cdot \frac{FOV}{2}\right)=E_{\mathrm{sat}}$$

Using simplification S2 and simplification S3, we get

(49) $$\frac{P_{\mathrm{laser},\max}T\lambda F}{\pi^{3}f^{3}}\cdot \frac{1}{\Theta_{\epsilon }{}^{3}}\cdot \frac{2}{\nu^{2}}\exp\left(-\frac{2}{\nu^{2}}\right)+\frac{P_{\mathrm{laser},\max}TN_{\mathrm{ss}}b_{0}}{f^{2}}\frac{1}{{\left(v^{*}\right)}^{2}}{\left[1+\frac{1}{{\left(v^{*}\right)}^{2}}{\left(\frac{\Theta_{\epsilon }}{l}\right)}^{2}\right]}^{\frac{s}{2}}\;\cdot \left(1-\exp\left(-\frac{2}{\nu^{2}}\right)\right)=E_{\mathrm{sat}}$$

which leads to:

(50) $$P_{\mathrm{laser},\max}=\frac{E_{\mathrm{sat}}f^{2}}{T}\frac{1}{\frac{\lambda F}{\pi^{3}f}\cdot \frac{1}{\Theta_{\epsilon }{}^{3}}\cdot \frac{2}{\nu^{2}}\exp\left(-\frac{2}{\nu^{2}}\right)+N_{\mathrm{ss}}b_{0}\frac{1}{{\left(v^{*}\right)}^{2}}{\left[1+\frac{1}{{\left(v^{*}\right)}^{2}}{\left(\frac{\Theta_{\epsilon }}{l}\right)}^{2}\right]}^{\frac{s}{2}}\cdot \left(1-\exp\left(-\frac{2}{\nu^{2}}\right)\right)}$$

The maximum dazzle exposure is then given by:

(51) $$MDE_{S}\left(\epsilon \right)=\frac{P_{\mathrm{laser},\max}}{\frac{\pi }{8}d_{86}^{2}}\;=\frac{8E_{\mathrm{sat}}f^{2}}{\pi d_{86}^{2}T}\frac{1}{\frac{\lambda F}{\pi^{3}f}\cdot \frac{1}{\Theta_{\epsilon }{}^{3}}\cdot \frac{2}{\nu^{2}}\exp\left(-\frac{2}{\nu^{2}}\right)+N_{\mathrm{ss}}b_{0}\frac{1}{{\left(v^{*}\right)}^{2}}{\left[1+\frac{1}{{\left(v^{*}\right)}^{2}}{\left(\frac{\Theta_{\epsilon }}{l}\right)}^{2}\right]}^{\frac{s}{2}}\cdot \left(1-\exp\left(-\frac{2}{\nu^{2}}\right)\right)}$$

Using Equation (44), we get:

(52a) $$MDE_{S}\left(\epsilon \right)=\frac{4E_{\mathrm{sat}}F^{2}}{\pi T}\frac{1}{\frac{\lambda F}{\pi^{3}f}\cdot \frac{1}{\Theta_{\epsilon }{}^{3}}\cdot \exp\left(-\frac{2}{\nu^{2}}\right)+N_{\mathrm{ss}}b_{0}\frac{1}{{\left(v^{*}\right)}^{2}}{\left[1+\frac{1}{{\left(v^{*}\right)}^{2}}{\left(\frac{\Theta_{\epsilon }}{l}\right)}^{2}\right]}^{\frac{s}{2}}\cdot \frac{\left(1-\exp\left(-\frac{2}{\nu^{2}}\right)\right)}{2/\nu^{2}\;}}$$

As in the case of the MPE_S_, the equation simplifies for the case of ν→∞, which gives the minimum value of the MDE_S_:

(52b) $$MDE_{S,\min}\left(\epsilon \right)=\underset{\nu \to \infty }{\lim}MDE_{S}\left(\epsilon \right)=\frac{4E_{\mathrm{sat}}F^{2}}{\pi T}\frac{1}{\frac{\lambda F}{\pi^{3}f}\cdot \frac{1}{\Theta_{\epsilon }{}^{3}}+N_{\mathrm{ss}}b_{0}{\left[1+{\left(\frac{\Theta_{\epsilon }}{l}\right)}^{2}\right]}^{\frac{s}{2}}}$$

Equations (52a) is a somewhat longer equation but is still a closed-form expression. Since we used simplification S3, which is neglecting the Gaussian approximation for the central lobe of the diffraction pattern, Equations (52a/b) should not be used for a value of ϵ=0 or very near to zero, see Appendix B.2 for more details. The value $MDE_{S}\left(\epsilon =0\right)$ would correspond to the onset of laser dazzle. This onset can be estimated by using Equation (45a) or (45b), but replacing the focal plane damage threshold $E_{\mathrm{dam}}$ by the saturation threshold $E_{\mathrm{sat}}$:

(53a) $$MDE_{S}\left(\epsilon =0\right)=E_{\mathrm{sat}}\cdot \frac{16\lambda^{2}F^{4}}{T\pi^{2}f^{2}}{\left[\frac{\frac{1}{\nu^{2}}}{1-\exp\left(-\frac{1}{\nu^{2}}\right)}\right]}^{2}$$

(53b) $$MDE_{S,\min}\left(\epsilon =0\right)=\underset{\nu \to \infty }{\lim}MDE_{S}\left(\epsilon =0\right)=E_{\mathrm{sat}}\cdot \frac{16\lambda^{2}F^{4}}{T\pi^{2}f^{2}}$$

For the calculation of the $MDE_{s}$ according to Equations (52a/b) and Equations (53a/b), the saturation threshold $E_{\mathrm{sat}}$ is needed. Section 5.3 gives more details on this quantity.

### 4.3. Hazard Distances

The calculation of the hazard distances for sensors follows the classical way to compute the hazard distances for the human eye according to the standards [43,44]:

(54) $$NSeHD=\frac{\sqrt{\frac{4P_{0}}{\pi \cdot MPE_{s}}-d_{0}^{2}}}{\Phi }\approx \frac{\sqrt{\frac{4P_{0}}{\pi \cdot MPE_{s}}}-d_{0}}{\Phi }$$

(55) $$NSeDD=\frac{\sqrt{\frac{4P_{0}}{\pi \cdot MDE_{s}}-d_{0}^{2}}}{\Phi }\approx \frac{\sqrt{\frac{4P_{0}}{\pi \cdot MDE_{s}}}-d_{0}}{\Phi }$$

The first term given in Equation (54) and (55) corresponds to ANSI Z136.6 [43] and utilizes Equation (5) to calculate the laser beam diameter: $d\left(z\right)=\sqrt{d_{0}^{2}+\Phi^{2}z^{2}}$. The second term is an approximation used in the German Technical Rules regarding the Artificial Optical Radiation Ordinance [44] utilizing a simplified calculation of the beam diameter: $d\left(z\right)=d_{0}+\Phi z$.

Equation (54) and (55) require the beam diameter $d_{0}$ and the divergence Φ to be defined according to the 1/e-intensity points of the beam profile, as it is standard for laser safety calculations. When using the 1/e^2^-definition for beam diameter and divergence, Equation (54) and Equation (55) has to be modified by replacing the factor 4 by a factor 8, see Equation (1).

Furthermore, Equation (54) and (55) are related to the laser output power $P_{0}$ and do not include atmospheric extinction. This can be incorporated by the approximations [44]:

(56) $$NSeHD_{\mu }=0.5\cdot NSeHD\cdot \left[1+\exp\left(-\mu \cdot NSeHD\right)\right]$$

(57) $$NSeDD_{\mu }=0.5\cdot NSeDD\cdot \left[1+\exp\left(-\mu \cdot NSeDD\right)\right]$$

### 4.4. Size of the Dazzle Spot

The size of a dazzle spot $\Theta_{\mathrm{dazzle}}$ can be calculated by equating the total irradiance $E_{\mathrm{fp}}\left(\Theta \right)$ of Equation (40) and the saturation irradiance $E_{\mathrm{sat}}$ and solving this equation for Θ:

(58) $$E_{\mathrm{fp}}\left(\Theta_{\mathrm{dazzle}}\right)=\eta_{d}E_{d}\left(\Theta_{\mathrm{dazzle}}\right)+E_{s}\left(\Theta_{\mathrm{dazzle}}\right)=E_{\mathrm{sat}}$$

Unfortunately, there is no closed form expression for this solution. This equation could be solved numerically using a computer. An approximate analytic solution can be given by solving the equations for the diffracted irradiance and the scattered irradiance individually and chose the maximum value as dazzle radius. Again, the simplifications S1–S3 will be applied.

Using simplification S2 and S3, the equation to solve for the diffracted irradiance $E_{d}$ is

(59) $$E_{d}\left(\Theta_{\mathrm{dazzle},d}\right)\approx E_{\mathrm{mean}}\left(\Theta_{\mathrm{dazzle},d}\right)=\frac{1}{\Theta_{\mathrm{dazzle},d}{}^{3}}\cdot \frac{2}{\nu^{2}}\exp\left(-\frac{2}{\nu^{2}}\right)=E_{\mathrm{sat}}$$

and the equation to solve for the scattered irradiance $E_{s}$ is

(60) $$E_{s}\left(\Theta_{\mathrm{dazzle},s}\right)=\frac{P_{\mathrm{laser}}TN_{\mathrm{ss}}b_{0}}{f^{2}}{\left[1+{\left(\frac{\Theta_{\mathrm{dazzle},s}}{l}\right)}^{2}\right]}^{\frac{s}{2}}\cdot \left(1-\exp\left(-\frac{2}{\nu^{2}}\right)\right)=E_{\mathrm{sat}}$$

Solving Equation (59) and (60) for Θ we get

(61) $$\Theta_{\mathrm{dazzle},d}=\sqrt[{3}]{\frac{P_{\mathrm{laser}}T}{E_{\mathrm{sat}}}\cdot \frac{\lambda F}{\pi^{3}f^{3}}\cdot \frac{2}{\nu^{2}}\exp\left(-\frac{2}{\nu^{2}}\right)}$$

(62) $$\Theta_{\mathrm{dazzle},s}=l\cdot \sqrt{{\left(\frac{E_{\mathrm{sat}}}{P_{\mathrm{laser}}T}\cdot \frac{f^{2}}{N_{\mathrm{ss}}b_{0}}\cdot \frac{1}{\left(1-\exp\left(-\frac{2}{\nu^{2}}\right)\right)}\right)}^{\frac{2}{s}}-1}$$

and finally

(63) $$\Theta_{\mathrm{dazzle}}\approx \max\left(\Theta_{\mathrm{dazzle},d},\;\Theta_{\mathrm{dazzle},s}\right)$$

## 5. Parameters for Laser Safety Calculations

In order to perform calculations according to the equations presented in the previous sections various parameters have to be known. Besides standard parameters for the laser source, the camera lens and the sensor given by the respective manufacturer, some further parameters are necessary, which are usually not specified/known. These are:

1. the scatter parameters of the camera lens,
2. the damage threshold of the imaging sensor and
3. the saturation threshold of the imaging sensor.

The following sections are attributed to these parameters. In Section 5.1, experimentally estimated values for the scatter parameters will be presented. Section 5.2 states typical values for the laser-induced damage threshold of CMOS and CCD cameras and Section 5.3 discusses a simple approach for calculating the saturation threshold of a sensor.

### 5.1. Scatter Parameters

The scatter parameters of various commercial off-the-shelf camera lenses were estimated. Very briefly, this was done by illuminating a camera lens with laser light and measuring the irradiance distribution in the focal plane using a CMOS camera. Since the camera’s dynamic range is not sufficient to measure the complete range of irradiance values occurring in the focal plane, a series of camera images was taken for each camera lens under test. For each image of the series, a different combination of laser power and camera integration time was used to map the irradiance distribution over the entire imaging sensor. Subsequently, a radial irradiance profile could be created from the camera images of the series. Finally, Equation (40) was fitted to the radial irradiance profile to estimate the scatter parameters. The measurements were performed at four different wavelengths (488 nm, 515 nm, 561 nm, and 640 nm). For the fit, the wavelength scaling laws of Equation (36) were considered.

Table 2 lists the camera lenses used for the experiments and their specifications. The camera lenses have different values of focal length, *f*-number and number of lenses. Intentionally, low-priced as well as higher-priced camera lenses were used in the experiments.

**Figure 11:**
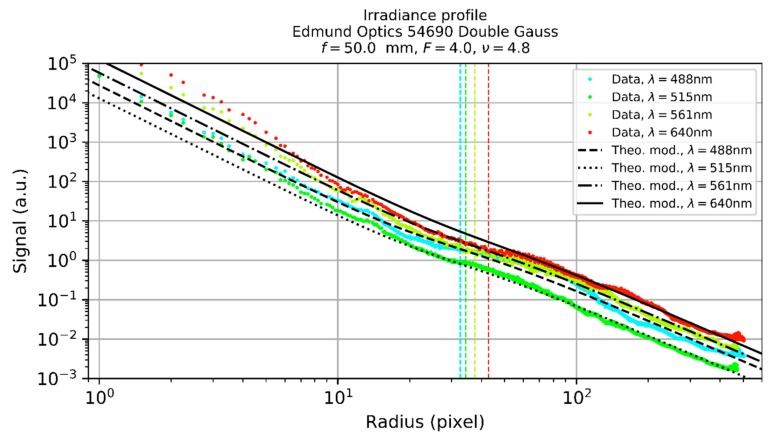
Typical irradiance profile in the focal plane of a camera lens. The vertical lines mark the scatter parameter l.

Figure 11 shows an example of typical irradiance profiles obtained by our measurements. In the graphs of Figure 11, the measured data is shown as colored dots, whereas the fits regarding the theoretical model are shown as black lines. The colored vertical lines shown in the graphs correspond to the values of the scatter parameter l. We can see from Figure 11 that the theoretical curves coincide quite well with the measurement data for radial coordinates larger than ~10 pixel.

The exact process of data acquisition and analysis is complex and will be described in more detail in a dedicated publication. Here, I will focus on the results to give the reader an idea what values should be used for the scatter parameters to perform the laser safety calculations. The results of the measurements are summarized in Table 3 for a reference wavelength of 550 nm. For each camera lens, the set of scatter parameters is not the result of a single measurement but of a multitude of measurements for different values of the truncation factor ν. In Table 3, the mean values for each camera lens are stated. Additionally, in the last lines the mean, median, standard deviation, and coefficient of variation of the tabulated values are given.

Looking at Table 3, we can see that the coefficient of variation (ratio of the standard deviation to the mean) is quite low for the scatter parameter s and acceptable for the for the scatter parameter l. This means that these scatter parameters, especially scatter parameter s, are quite similar for all the camera lenses tested. However, for the scatter parameter $b_{0}$, the coefficient of variation is larger than one. Therefore, it may be that no single value can be stated for $b_{0}$ that will fit all camera lenses.

Please note that I am still in the process of measuring the scatter parameters to improve the statistical database. Nevertheless, the table should offer good reference values for scatter parameters of typical camera lenses. Based on these preliminary results, I propose to use the median values of the scatter parameters given by Table 3, if measured values are not available:

$$s=-2.5,\;b_{0}=1.2\;{\mathrm{sr}}^{-1},\;l=5.3\cdot 10^{-3}\;\mathrm{rad}\;\mathrm{for}\;\lambda_{0}=550\;\mathrm{nm}$$

### 5.2. Laser Damage Threshold

Information on laser induced damage thresholds for continuous-wave (cw) laser radiation in the visible spectral range are rare. Schwarz and co-workers measured cw laser induced damage thresholds (LIDT) of CCD and CMOS cameras for a wavelength of 532 nm [28]. Here, I refer to this publication and summarize that threshold values in Table 4. Please note that Schwarz measured these values for specific imaging sensors (CCD sensor: Sony ICX098, CMOS sensor: Aptina MT9V024). Laser damage thresholds for other types of imaging sensors may vary; but the order of magnitude (10–100 kW/cm^2^) should be appropriable. Bartoli and co-workers measured damage thresholds of the same order of magnitude for a silicon detector at a laser wavelength of 690 nm [45].

### 5.3. Laser Saturation Threshold

The saturation threshold of an imaging sensor may be calculated using its technical specifications. We assume that a pixel of the imaging sensor is illuminated with irradiance E (W/m^2^). The number of photons $\mu_{p}$ arriving at the pixel of area A (m^2^) during the exposure time $t_{\exp}$ (s) is given by

(64) $$\mu_{P}=\frac{EAt_{exp}}{hc/\lambda }$$

where $h=6.626\cdot 10^{-34}\;\mathrm{Js}$ is the Planck constant and $c=2.99792458\cdot 10^{8}\frac{m}{s}$ the speed of light in a vacuum. The number of photoelectrons $\mu_{e}$ generated in the pixel is determined by the (wavelength-dependent) quantum efficiency η:

(65) $$\mu_{e}=\eta \cdot \mu_{p}$$

At a specific irradiance $E_{\mathrm{sat},\mathrm{pixel}}$ (i.e., the saturation irradiance), the number of photoelectrons $\mu_{e}$ will equal the saturation capacity of a pixel. Generally, the saturation capacity is lower than the full well capacity C of the pixel [46]. For simplicity, we equal these values. The saturation irradiance of a pixel can then be estimated by

(66) $$C=\mu_{e}=\eta \cdot \mu_{p}=\eta \cdot \frac{E_{\mathrm{sat},\mathrm{pixel}}At_{\exp}}{\frac{hc}{\lambda }}$$

resulting in

(67) $$E_{\mathrm{sat},\mathrm{pixel}}=\frac{C\cdot hc/\lambda }{\eta At_{\exp}}$$

If the full well capacity for the imaging sensor in use is not known, it may be estimated by a rule of thumb stated by Holst and Lomheim [47]:

(68) $$C=1500\frac{e^{-}}{{\mu m}^{2}}\cdot A$$

The area A of one pixel can be calculated from pixel pitch p (m) and fill factor ff:

(69) $$A=p^{2}\cdot ff$$

If the fill factor is not known, we set ff=1.

Putting just the pixel saturation irradiance $E_{\mathrm{sat},\mathrm{pixel}}$ from Equation (67) into the MDE_S_ equations Equations (52a/b) and (53a/b) would imply that the sensor is illuminated by the dazzle laser only. In a real situation, the sensor also observes a scene, which means that the capacity of a pixel is utilized by the light of the scene and the laser light. Usually, the operator or the camera’s automatic exposure (AE) control will set the exposure time to a level that the mean pixel value of the sensor image equals roughly half of the maximum pixel value. Thus, applying a factor of 0.5 to Equation (67), we get an estimate for the saturation irradiance:

(70) $$E_{\mathrm{sat}}\approx 0.5\cdot E_{\mathrm{sat},\mathrm{pixel}}=0.5\cdot \frac{C\cdot hc/\lambda }{\eta At_{\exp}}$$

For cases where the mean pixel value ($pv_{\mathrm{mean}}$) of a scene is explicitly known, one could use a factor $1-\frac{pv_{\mathrm{mean}}}{pv_{\max}}$ instead of 0.5. The maximum pixel value $pv_{\max}$ is given by the sensor’s bit depth bd: $pv_{\max}=2^{bd}-1$. Thus, the saturation irradiance can be calculated by:

(71) $$E_{\mathrm{sat}}\approx \left(1-\frac{pv_{\mathrm{mean}}}{pv_{\max}}\right)\cdot \frac{C\cdot hc/\lambda }{\eta At_{\exp}}$$

As we will see in Section 6.2, Equation (71) is good for specialists working in a laboratory environment, when the mean pixel value or the target grey value of the AE control is known. Otherwise, Equation (70) would be appropriate.

## 6. Calculation Examples

### 6.1. Example 1: Calculation of MPE_S_ and NSeHD for a Monochrome CMOS Sensor

Schwarz et al. measured the laser induced damage threshold (LIDT) of a CMOS camera (imaging sensor Aptina MT9V024) for continuous-wave laser radiation [28]. The experimental parameters and the results were as follows:

1. Laser: Laser Quantum Ventus 532 (continuous-wave)
  (1) Maximum laser output power: $P_{0}>500\;\mathrm{mW}$
  (2) Wavelength: λ=532 nm
  (3) Beam diameter at the camera lens: (1/e^2^) $d_{86}=3\;\mathrm{mm}$
2. Camera lens: Qioptiq Apo-Rodagon-N 4.0/80
  (1) Focal length: f=80 mm
  (2) *f*-number: F=5.6
  (3) No. of optical elements: $N_{\mathrm{oe}}\leq 8$ (the specification states up to 8 lenses for the lenses of the Apo-Rodagon series, depending on the focal length)
  (4) Focal spot diameter (1/e^2^): $d_{\mathrm{spot}}=25.7\;µm$
3. Results for 1 s exposure:
  (1) Occurrence of damage observed at a focal peak irradiance of 85 kW/cm^2^
  (2) Estimated LIDT (for 1 s exposure): $E_{\mathrm{dam}}=73\frac{\mathrm{kW}}{{\mathrm{cm}}^{2}}$

Based on these experimental parameters, I want to calculate the MPE_S_ and NSeHD for the CMOS camera.

#### 6.1.1. MPE_S_

For the experimental setup of Schwarz et al., the truncation factor is

$$\nu =\frac{d_{86}}{d_{\mathrm{ap}}}=\frac{d_{86}F}{f}=\frac{3.0\;\mathrm{mm}\;\cdot 5.6}{80\;\mathrm{mm}}=0.21$$

Since the transmittance of the camera lens is not specified, I estimate it as follows: I assume that the lens has 7 optical elements, $N_{\mathrm{oe}}=7$, which means that the number of scattering surfaces is $N_{\mathrm{ss}}=14$. I further assume that all optical elements are coated with a broadband anti-reflection coating (BBAR) having a reflectance below 0.5 percent. Thus, the lens transmittance results in

$$T={\left(1-0.005\right)}^{N_{\mathrm{ss}}}=0.995^{14}=0.93$$

and subsequently the maximum permissible exposure of the sensor for ν=0.21 is according Equation (45a)

$$MPE_{S}=E_{\mathrm{dam}}\cdot \frac{16\lambda^{2}F^{4}}{T\pi^{2}f^{2}}{\left[\frac{\frac{1}{\nu^{2}}}{1-\exp\left(-\frac{1}{\nu^{2}}\right)}\right]}^{2}=2.8\frac{W}{{\mathrm{cm}}^{2}}$$

From this result, we can calculate the maximum power of the incident laser beam to be safe from sensor damage:

$$P_{\mathrm{laser},\max}=MPE_{S}\frac{\pi d_{86}^{2}}{8}=99\;\mathrm{mW}$$

Schwarz et al. observed the occurrence of damage at a focal plane irradiance of 85 kW/cm^2^. This corresponds to an incident laser power of (see Equation (2))

$$P_{\mathrm{damage}}=85\frac{\mathrm{kW}}{{\mathrm{cm}}^{2}}\cdot \frac{\pi }{8}d_{\mathrm{spot}}^{2}\;/\;T=237\;\mathrm{mW}$$

which is, as expected, higher than the laser power of 99 mW corresponding to the MPE_S_.

#### 6.1.2. NSeHD

If we want to calculate the hazard distance NSeHD for the laser source used by Schwarz et al., we need some additional information. Reference [28] does not state the beam diameter at the laser exit port and the beam divergence since it is not of relevance for these experiments. However, these values were measured to be:

- Divergence (1/e^2^): Φ=0.55 mrad
- Beam diameter at the laser exit port (1/e^2^): $d_{0}=1.25\;\mathrm{mm}$

For the calculation of the NSeHD, we consider the worst-case scenario and calculate the minimum value of the MPE_S_ according to Equation (45b):

$$MPE_{S,\min}=E_{\mathrm{dam}}\cdot \frac{16\lambda^{2}F^{4}}{T\pi^{2}f^{2}}=5.5\frac{\mathrm{mW}}{{\mathrm{cm}}^{2}}$$

Using this value, we can estimate the hazard distance using Equation (54), whereby the values of beam diameter and divergence have to be defined according to the 1/e-intensity points of the beam profile. This means that the values $d_{0}$ and Φ stated above have to be divided by $\sqrt{2}$ for the calculation, which results in:

$$NSeHD=\frac{\sqrt{\frac{4P_{0}}{\pi \cdot MPE_{s,\min}}-{\left(\frac{d_{0}}{\sqrt{2}}\right)}^{2}}}{\Phi /\sqrt{2}}=276\;m$$

### 6.2. Example 2: Calculation of Dazzle Spot Size and MDE_S_

In previous work, we performed laser dazzle experiments on a sensor system hardened against laser dazzle [11]. In the frame of those experiments, the vulnerability to laser dazzle of this hardened sensor was compared to standard CMOS cameras (monochrome and color). Briefly, the sensors observed a highly structured fractal test chart [48], consisting of a multitude of dark and bright quadrats. Using a multi-wavelength laser source, the sensors were irradiated with laser light of different wavelength and power. From the image data acquired during the experiments, the loss of image information due to laser dazzle was assessed as a function of laser power and wavelength. I will not go into more details of this work, but refer the reader to the aforementioned publication [11].

In this calculation example, I used the experimental parameters regarding two monochrome CMOS cameras and calculated dazzle spot sizes according Section 4.4. The result of the calculation is compared to the image data gathered with the two monochrome CMOS camera during the experiments. Furthermore, I used the image data to determine the corresponding dazzle level ϵ for each camera image. Using the values of the dazzle level, the respective MDE_S_ values were calculated. The MDE_S_ values are compared to measured values of laser irradiance $E_{\mathrm{laser}}$ in front of the camera lens. In this calculation example, I will refrain from calculating the NSeDD, since the procedure is exactly the same as in Section 6.1.2 for the NSeHD.

The parameters used for the calculation of dazzle spot sizes are given in Table 5. Since the scatter parameters of these camera lenses are not available, I applied the values proposed in Section 5.1 of s=−2.5, $b_{0}=1.2\;{\mathrm{sr}}^{-1}$ and $l=5.3\cdot \times \;10^{-3}\;\mathrm{rad}$ for a reference wavelength of $\lambda_{0}=550\;\mathrm{nm}$. The wavelength scaling laws of Equation (36) were applied to them. I started the calculations with the estimation of the wavelength-dependent saturation irradiance for both cameras using Equation (71), see Table 6 for the results.

#### 6.2.1. Dazzle Spot Size

For each wavelength, the CMOS cameras were dazzled using 24 different values of input power. Dazzle spot sizes were calculated for all wavelengths and the different input powers. Using Equation (61) and (62), I calculated the dazzle spot sizes for the two cases of diffraction only $\Theta_{\mathrm{dazzle},d}$ and scatter only $\Theta_{\mathrm{dazzle},s}$. Additionally, I numerically solved Equation (58) for Θ to get an exact solution $\Theta_{\mathrm{num}}$ for the dazzle spot size. Here, the word “exact” is related to the solution of Equation (58), not to claim that the Equation (58) itself is a perfect description of reality.

As an example, Table 7 shows the results for Setup 2 and a laser wavelength of 488 nm. The first column of the table contains the laser irradiance in front of the camera lens as measured during the experiment ($E_{\mathrm{laser}}$). In the second column, calculated values of the laser power entering the camera lens are shown ($P_{\mathrm{in}}$). The columns 3–5 present the three different calculated dazzle spot sizes ($\Theta_{\mathrm{dazzle},d}$, $\Theta_{\mathrm{dazzle},s}$, $\Theta_{\mathrm{num}}$). Results for Setup 1 (laser wavelength 488 nm and 640 nm) and further results for Setup 2 (laser wavelength 640 nm) can be found in Appendix C, Table A2, Table A3 and Table A4.

**Figure 12:**
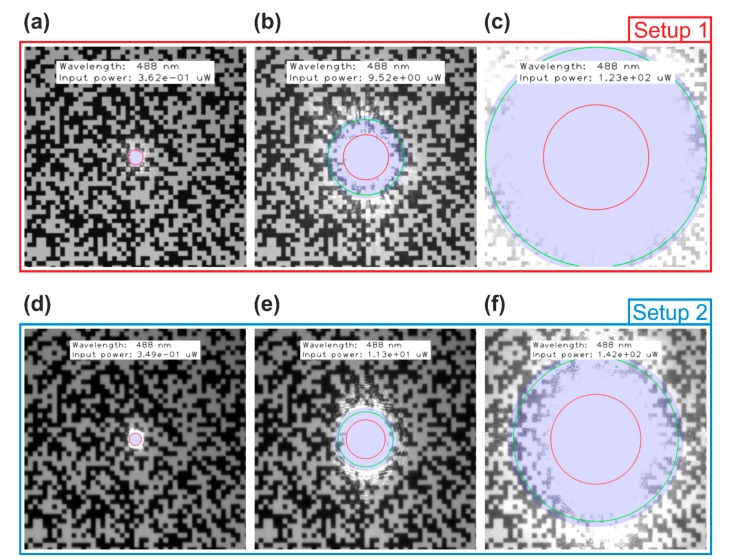
Laser dazzle spots recorded with two monochrome CMOS camera for a laser wavelength of 488 nm: (**a**–**c**): VR magic VRmC-12/BW Pro and (**d**–**f**) Allied Vision Mako G-158B. Additionally, calculated spot sizes are drawn into the camera images: Red circle: spot size for diffraction only, green circle: spot size for scatter only, blue disk: spot size taking diffraction and scatter into account.

**Figure 13:**
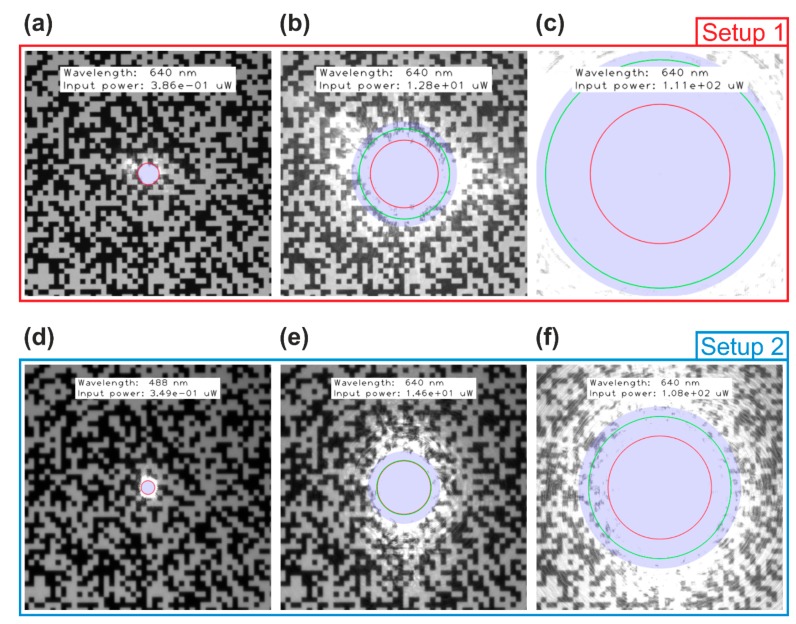
Laser dazzle spots recorded with two monochrome CMOS camera for a laser wavelength of 640 nm: (**a**–**c**): VRmagic VRmC-12/BW Pro and (**d**–**f**) Allied Vision Mako G-158B. Additionally, calculated spot sizes are drawn into the camera images: Red circle: spot size for diffraction only, green circle: spot size for scatter only, blue disk: spot size taking diffraction and scatter into account.

For illustration, I overlaid the different calculated dazzle spot sizes with the camera images. As a selection, six images are shown for each camera, three for the wavelength 488 nm and three for the wavelength 640 nm. Figure 12 shows camera images with dazzle spots of different size (due to different laser power) using a laser wavelength of 488 nm. Figure 13 is similar but for a laser wavelength of 640 nm. In both figures, the calculated dazzle spot sizes are plotted into the camera images. The blue transparent disk corresponds to the numerical solution $\Theta_{\mathrm{num}}$. The approximations are drawn as colored circles. Red and green circles correspond to $\Theta_{\mathrm{dazzle},d}$ (diffraction only) and $\Theta_{\mathrm{dazzle},s}$ (scatter only), respectively. The calculated values corresponding to the images of Figure 12; Figure 13 are printed in bold face in Table 7.

From Figure 12, Figure 13 we can conclude:

1. For very small dazzle spots (Figure 12a,d, Figure 13a,d), the dazzle spot size can be approximated by just using only the diffraction part. The red circle corresponds to the edge of the blue disk.
2. For large dazzle spots (Figure 12c,f), the dazzle spot size cannot be estimated assuming diffraction only. As stated in Section 3.4, the main contribution to the dazzle spot is scattered light. The green circle coincides roughly with the edge of the blue disk.
3. When the diffraction and the scatter contribution are nearly equal (Figure 13e), there is some difference of the spot sizes for diffraction and scatter only to the numerically calculated dazzle spot size, but it keeps within limits.

Additionally, the measurement data was analyzed by the method of overexposed pixels counting (OPC). For more details on this method see, e.g., reference [14] or [19]. Briefly, the number of overexposed pixels in a camera image is counted and the size of a disk (diameter or radius) containing the same amount of pixels is calculated. This quantity quite well represents the size of the dazzle spot. The measured dazzle spot sizes are also contained in Table 7 for comparison.

Overall, I expect that the approximation of Equation (63) for the dazzle spot size is reasonable. The calculated dazzle spot sizes coincide quite well with the experimental data considering that the scatter parameters were estimated employing a different camera and different camera lenses (see Table 2).

#### 6.2.2. MDE_S_

Using the experimentally estimated dazzle spot sizes, the dazzle level ϵ for each camera image was calculated by applying Equation (46). Subsequently, MDE_S_ values were calculated according Equation (52a) for these dazzle levels. The calculated values of dazzle level and MDE_S_ are also given in Table 7; Table A2, Table A3 and Table A4 for the different setups and laser wavelengths.

Ideally, the calculated values of MDE_S_ would correspond to the measured values of the laser irradiance $E_{\mathrm{laser}}$ in front of the camera lens. We can see that the difference between these two quantities is within a factor of 2 except for very low dazzle levels. In Appendix B, I discuss the limits of applicability of the derived equations. According to Equation (A8), the value of dazzle level ϵ should be chosen greater than 0.04 for MDE_S_ calculations for both setups. This means that we cannot demand that the calculated values of dazzle spot size in Table 7 match the measured values for the dazzle levels of 0.04 and lower.

### 6.3. Comparison of MPE_S_ and MDE_S_

Regarding the MPE and MDE for the human eye, it is known that the value of MDE can be higher than the value of MPE under certain conditions (e.g., MDE for a high dazzle level at daylight) [26]. This leads to a scenario where the hazard distance for eye dazzle (NODD) is shorter than the hazard distance for eye damage (NOHD), in contrast to the situation shown in Figure 1.

One would not expect such an effect for imaging sensors, since from experience it is known that a camera sensor usually can be dazzled completely (all sensor pixel saturated) without being damaged. If we compare the Equation (45a) for the MPE_S_ and Equation (53a) describing the onset of sensor dazzle MDE_S_ (ε = 0), we can see that such a situation could only occur when the saturation irradiance $E_{\mathrm{sat}}$ of a sensor is greater than its damage threshold $E_{\mathrm{dam}}$. Here, I will discuss if this is possible and what the prerequisites would be.

Using Equation (70), the condition $E_{\mathrm{sat}}>E_{\mathrm{dam}}$ leads to the equation

(72) $$E_{\mathrm{sat}}\approx 0.5\cdot \frac{C\cdot \frac{hc}{\lambda }}{\eta At_{\exp}}>E_{\mathrm{dam}}$$

The damage threshold $E_{\mathrm{dam}}$ of an imaging sensor depends on the laser wavelength λ and laser pulse duration τ, but is a fixed value for a specific scenario (i.e., a specific laser device). Most of the parameters used in Equation (70) to estimate the saturation threshold $E_{\mathrm{sat}}$ are also fixed values, which depend on the specific model of imaging sensor. The only parameter of Equation (72) that can be changed by the camera operator is the exposure time $t_{\exp}$. Therefore, we solve this equation for $t_{\exp}$ and get

(73) $$t_{\exp}<0.5\cdot \frac{C\cdot \frac{hc}{\lambda }}{\eta AE_{\mathrm{dam}}}$$

as condition for a scenario, where $E_{\mathrm{sat}}$ is greater than $E_{\mathrm{dam}}$. This would lead to a situation where the hazard distance for sensor damage NSeHD is larger than the hazard distance for sensor dazzle NSeDD, in contrast to the situation shown in Figure 9.

For a sample calculation, I use the parameters of Setup 1 (see Table 5), assume a laser wavelength of 550 nm and take the value $E_{\mathrm{dam}}=73\frac{\mathrm{kW}}{{\mathrm{cm}}^{2}}$ stated by Schwarz et al. [28] as laser damage threshold for a CMOS camera illuminated with continuous-wave laser radiation. The condition $E_{\mathrm{sat}}>E_{\mathrm{dam}}$ would be fulfilled for:

$$t_{\exp}<0.5\cdot \frac{C\cdot \frac{hc}{\lambda }}{\eta AE_{\mathrm{dam}}}=\frac{0.5\cdot 6000\cdot hc}{550\;\mathrm{nm}\cdot 0.48\cdot {\left(6\;µm\right)}^{2}\cdot 73\frac{\mathrm{kW}}{{\mathrm{cm}}^{2}}}=8.6\cdot 10^{-14}\;s$$

This is a quite short exposure time. Typically, standard cameras are operated with exposure times ranging from some microseconds up to several tens of milliseconds. I would therefore assume that the situation described above will not occur for imaging sensors.

## 7. Conclusions

In this publication, an approach to perform laser safety calculations for camera sensors was presented. This is the first time that such investigations have been carried out for this purpose. The derived physical quantities like Maximum Permissible Exposure for a Sensor MPE_S_ and Maximum Dazzle Exposure for a Sensor MDE_S_ were defined according to the already existing respective quantities for the human eye (MPE, MDE). Therefore, the calculation of corresponding hazard distances like Nominal Sensor Hazard Distance (NSeHD) and Nominal Sensor Dazzle Distance (NSeDD) can be performed in exactly the same way as it is done for the human eye. I focused my attention on providing closed-form equations in order to enable users who are not experts in this field to perform these laser safety calculations. However, it was a kind of a tightrope act to reduce the equations to as simple forms as possible, keeping a sufficient description of reality at the same time.

In a first step, I started deriving closed-form equations in order to describe the focal plane irradiance pattern generated by the camera lens, consisting of diffraction and scatter parts. To reduce complexity of the equations, aberrations of the camera lens were neglected. This is reasonable since aberrations cause a reduction of the peak irradiance and distribute the power over a larger area at the focal plane, thus giving more safety to this approach. Subsequently, the laser safety quantities MPE_S_ and MDE_S_ were derived using the equations for the focal plane irradiance distribution. In addition, the limits of the approach regarding applicability were examined.

The equations describing the focal plane irradiance distribution or the laser safety quantities comprise mainly parameters, which are usually specified by the manufacturers of the camera lens and of the imaging sensor. However, some of the used parameters are typically not known. Primarily, these are the three parameters s, b and l used in the Harvey scatter model to estimate the stray light irradiance at the focal plane. Furthermore, values representing the laser-induced damage threshold (LIDT) and saturation irradiances for imaging sensors are normally not known. In order to help out, LIDT values for commercial off-the-shelf CCD and CMOS cameras were stated that were taken from literature and an approach to calculate saturation irradiances was presented. In order to gain own information on lens scattering, an experimental setup to measure the scatter parameters was established. This publication provides preliminary results in order to enable the envisaged laser safety calculations. Today, it is not yet clear whether a single set of scatter parameters is sufficient to describe the scattering of light by a typical camera lens. However, I am quite confident that this will be the case at least for the two scatter parameters s and l. Calculated values of dazzle spot sizes based on these first results are in good agreement with measured data from previous work.

Besides the estimation of the scatter parameters, future work on this topic should definitely include an extensive validation of the derived equations. This may be supported by a field trial to collect a large amount of data using a variety of sensors and camera lenses. Furthermore, a deeper examination of the derived equations is also of interest. Which parameters influence the value of MPE_S_/MDE_S_ in particular? Are there unexpected effects? As an example, in Section 6.3, I examined whether the MPE_S_ could be greater than the MDE_S_. The derived equations may give rise to other unexpected effects that need to be investigated and validated. Of great interest is the question of whether the equations can be further simplified without losing accuracy.

## Figures and Tables

**Table 1:** Numerically calculated values of the point of intersection $r_{\mathrm{pi}}$ of the mean irradiance according Equation (17) and the Gaussian approximation of the central lobe according Equation (18) for different values of wavelength λ, *f*-number F and truncation factor ν. Additionally, the spot radius $d_{\mathrm{spot}}/2$ according Equation (19) is listed for comparison.

λ (nm)	F	ν	$r_{\mathrm{pi}}=f\Theta_{\mathrm{pi}}\;(µm)$	$d_{\mathrm{spot}}/2\;(µm)$
445	2.8	0.3	9.7	2.6
		1.0	1.8	1.1
		2.0	1.4	1.0
	11.0	0.3	37.9	10.4
		1.0	7.1	4.3
		2.0	5.5	4.0
532	2.8	0.3	11.5	3.2
		1.0	2.2	1.3
		2.0	1.7	1.2
	11.0	0.3	45.3	12.4
		1.0	8.5	5.2
		2.0	6.6	4.8
635	2.8	0.3	13.8	3.8
		1.0	2.6	1.6
		2.0	2.0	1.4
	11.0	0.3	54.1	14.8
		1.0	10.1	6.2
		2.0	7.8	5.7

**Table 2:** Parameters of the investigated camera lenses.

Camera Lens.	Focal Length	*f*-Number	No. of Lenses	Coating	Price (approx.)
LINOS MeVis-C 1.8/50	50.6	1.8	7	unk.	700 €
Edmund Optics 54690 (Double Gauss)	50	4.0	6	MgF2	500 €
Edmund Optics 67715	25	1.4	7	BBAR	500 €
Edmund Optics 86410	100	2.8	7	BBAR	500 €
Schneider-Kreuznach Xenoplan 2.8/50	50.2	2.8	6	unk.	630 €
Navitar NMV-75	75	2.5	5	unk.	185 €
Navitar NMV-100	100	2.8	5	unk.	170 €

**Table 3:** Scatter parameters of the camera lenses to be used in the 3-paramater Harvey scatter model, related to a reference wavelength of 550 nm.

Camera Lens	Scatter Parameters		
	s	$b_{0}\;({\mathrm{sr}}^{-1})$	l (rad)
LINOS MeVis-C 1.8/50	−2.50	1.18	5.29 × 10^−03^
Edmund Optics 54690 (Double Gauss)	−2.54	5.83	3.81 × 10^−03^
Edmund Optics 67715	−2.14	0.92	7.43 × 10^−03^
Edmund Optics 86410	−2.64	10.81	4.09 × 10^−03^
Schneider-Kreuznach Xenoplan 2.8/50	−2.29	3.36	5.15 × 10^−03^
Navitar NMV-75	−2.39	0.49	5.46 × 10^−03^
Navitar NMV-100	−2.45	0.12	5.56 × 10^−03^
Mean	−2.42	3.24	5.25 × 10^−03^
Median	−2.45	1.18	5.29 × 10^−03^
Standard deviation	0.17	3.89	1.18 × 10^−03^
Coefficient of variation	0.07	1.20	0.22

**Table 4:** 1-on-1 Laser induced damage threshold (LIDT) measured for some specific imaging sensors (CCD: Sony ICX098, CMOS: Aptina MT9V024) [28].

1-on-1 LIDT (kW/cm^2^)				
Imaging Sensor	Exposure Time (s)			
	0.25	1	5	10
CMOS, monochrome	75 ± 7	73 ± 15	56 ± 4	48 ± 3
CMOS, color	56.7 ± 1.8			
CCD, monochrome	146 ± 9	118 ± 9	93 ± 19	95 ± 21
CCD, color	14 ± 2	13 ± 2	11 ± 1	8.1 ± 0.8

**Table 5:** Parameters used for the calculation of dazzle spot sizes.

Parameter	Setup 1	Setup 2
Camera	VRmagic VRmC-12/BW-Pro	Allied Vision Mako G-158B
Imaging sensor	Aptina MT9V024	Sony IMX273
No. of pixels	754 × 480	1456 × 1088
Quantum efficiency η	0.48 (all wavelengths)	0.63 (488, 515, 561 nm) 0.56 (640 nm)
Pixel size p (µm)	6	3.45
Fill factor ff	ukn.=1	ukn.=1
Exposure time $t_{\exp}$ (ms)	8.3	8.3
Maximum pixel value $pv_{\max}$	255	255
Saturation capacity C (e-)	6000	10500
Camera lens	Schneider-Kreuznach Apo-Xenoplan 2.0/35-2001	Kowa LM25NC3
Focal length f (mm)	35.1	25
f-number F	2	1.8
No. of optical elements $N_{\mathrm{oe}}$	7	7
Laser	Toptica iChrome MLE-L	
Wavelength λ (nm)	488 / 515 / 561 / 640	
Maximum laser power $P_{in}$ (mW)	1.6 / 0.7 / 1.6 / 1.0	0.96 / 0.42 / 0.89 / 0.61
Beam diameter $d_{86}$ (cm)	16.8 / 16.6 / 15.9 / 16.0	
Test chart	Fractal test chart [48]	
Mean pixel value $pv_{\mathrm{mean}}$	93	63

**Table 6:** Focal plane saturation irradiance calculated according to Equation (71).

$\mathrm{Focal}\;\mathrm{Plane}\;\mathrm{Saturation}\;\mathrm{Irradiance}\;E_{\mathrm{sat}}\;(µW/{\mathrm{cm}}^{2})$				
Sensor	Wavelength λ			
	488 nm	515 nm	561 nm	640 nm
VRmagic VRmC-12/BW-Pro	1.11	1.03	0.96	0.79
Allied Vision Mako G-158B	5.24	4.81	4.42	4.43

**Table 7:** Calculated and measured dazzle spot size using Setup 2 for a laser wavelength of 488 nm. Abbreviation: n.m.: not measurable.

$E_{\mathrm{laser}}\;(µW/{\mathrm{cm}}^{2})$	$P_{\mathrm{in}}\;(µW)$	Calculated Dazzle Spot Radius (pixel)			Measured Dazzle Spot Radius (pixel)	Dazzle Level ϵ	MDE_S_ (µW/cm^2^)
		$\Theta_{\mathrm{dazzle},d}$	$\Theta_{\mathrm{dazzle},s}.$	$\Theta_{\mathrm{num}}$			
2.78 × 10^−04^	4.20 × 10^−04^	2	0	2	3	0.004	1.94 × 10^−03^
3.19 × 10^−03^	4.82 × 10^−03^	4	0	4	13	0.02	0.107
4.72 × 10^−02^	7.13 × 10^−02^	9	0	10	18	0.02	0.263
0.143	0.215	14	0	14	21	0.03	0.377
**0.231**	**0.349**	**16**	**0**	**17**	**23**	**0.03**	**0.451**
0.667	1.01	23	0	27	29	0.04	0.771
1.67	2.53	31	27	42	44	0.06	1.92
3.75	5.66	40	50	60	64	0.09	4.22
**7.48**	**11.3**	**51**	**72**	**82**	**80**	**0.11**	**7.12**
9.42	14.2	55	80	90	85	0.12	8.15
11.9	17.9	59	89	99	91	0.13	9.67
14.9	22.5	64	99	109	97	0.13	11.3
23.7	35.7	75	121	132	111	0.15	15.6
29.8	45.0	81	134	145	121	0.17	19.3
37.5	56.6	87	148	159	131	0.18	23.0
48.3	72.9	95	164	176	153	0.21	34.0
56.7	85.7	100	176	188	169	0.23	43.6
66.7	101	105	188	200	186	0.26	55.5
**94.2**	**142**	**118**	**216**	**230**	**222**	**0.31**	**87.1**
119	179	128	238	252	260	0.36	129
149	225	138	261	276	298	0.41	182
358	541	185	372	389	475	0.65	596
461	696	201	412	430	n.m.	n.m.	-
637	961	224	469	488	n.m.	n.m.	-

**Figure A1:**
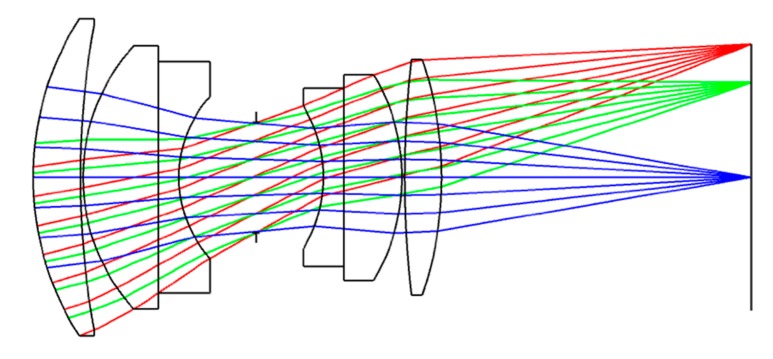
Beam path of a Double Gauss lens.

**Figure A2:**
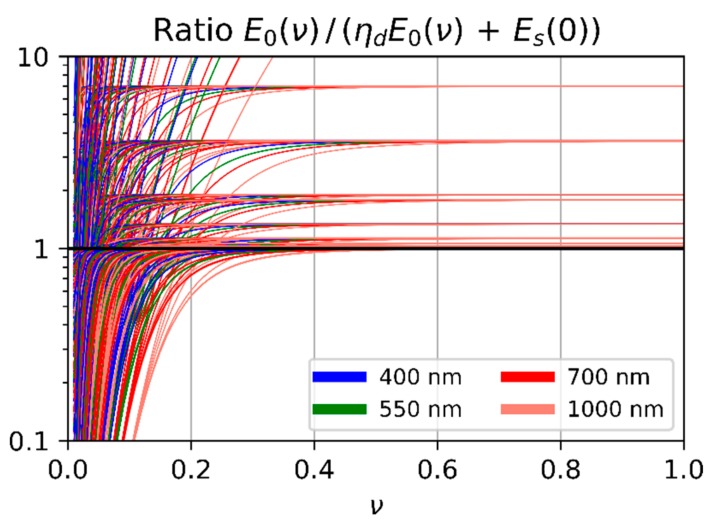
Deviation of the focal plane peak irradiance by using simplification S1 and simplification S2.

## Appendix A. Comment on Camera Lenses

### Approximation 2 used in Equation (30) Includes Two Simplifications

1. The size of the beam at the different scattering surfaces of the camera lens is equal to the size of the entering beam.
2. The size of the beam at the different scattering surfaces of the camera lens is constant.

Here, I want to discuss in more detail the rationale behind this approximation.

Manufacturers of camera lenses will usually not disclose the exact optical design of their products. Furthermore, the aim is to come to a closed-form expression for the focal plane irradiance, which is exact enough for laser safety calculations but as simple as possible. According to reference [49] “35-mm SLR normal lenses are invariably Double-Gauss types”. As an example, Figure A1 shows the beam path for such a Double Gauss with a focal length of f=100 mm and *f*-number of F=3. Such Double-Gauss lenses usually have 6–8 optical elements, see, for example, reference [50]. The camera lenses used for our stray light measurements comprise 5–7 optical elements (see Section 5.1). This means that Equation (29) for the scatter irradiance would contain 10–16 terms, which would prevent practical operability. Therefore, Equation (29) has to be simplified, as is done by Approximation 2 of Equation (31).

In principle, Approximation 2 could be modified by including a mean beam radius:

(A1) $$a_{j}=\bar{a_{j}}=k\cdot a_{\mathrm{ent}}=\mathrm{const}.$$

The factor k would then be another fit parameter in the data analysis of the stray light measurements presented in Section 5.1. I investigated the beam radii of 16 optical design examples for Double-Gauss lenses given by Smith [50]. For these optical designs, the mean beam radius at the optical elements is around 80 percent of the incident beam radius, i.e., k≈0.8. However, this factor k can be omitted, because it is included indirectly in the scatter parameters s, b and l by the fit of the theoretical model of Equation (35) to the data.

The same applies to the different contributions of stray light by the single scattering surfaces. In principle, according to Equation (29), each scattering surface would have a smaller or larger contribution depending on the beam radius $a_{j}$. But by fitting the simplified Equation (35) to the measurement data (see Section 5.1), this will finally be contained within the estimated scatter parameters.

We just have to accept the fact that the estimated scatter parameters for the camera lenses as a whole will not correspond to the scatter parameters of the single scattering surfaces. They also may have values that are not within the typical limits of $0.01\;{\mathrm{sr}}^{-1}\leq b\leq 1\;{\mathrm{sr}}^{-1}$ and −3≤s≤−1 (see Table 3).

## Appendix B. Limits of Applicability

### Appendix B.1. Applicability of Simplification S1 and Simplification S2

To derive an equation for the Maximum Permissible Exposure for a sensor MPE_S_, I used simplification S1 and simplification S2: $E_{\mathrm{fp}}\left(0\right)=\eta_{d}E_{0}\left(\nu \right)+E_{s}\left(0\right)\approx E_{0}\left(\nu \right)$.

While simplification S1 (neglecting the scattered light to estimate the focal plane peak irradiance) decreases the peak irradiance, simplification S2 (neglecting the reduction of diffracted laser power due to the scattered light) increases the peak irradiance. For laser safety calculations it is required that the focal plane peak irradiance is not underestimated. Underestimation would lead to a too low value of MPE_S_. An overestimation of the peak irradiance can be treated as a safety factor.

I examined the applicability of these two simplifications by calculating the ratio $E_{0}\left(\nu \right)/\left(\eta_{d}E_{0}\left(\nu \right)+E_{s}\left(0\right)\right)$ for a range of parameters and plotted the ratio as a function of truncation factor ν, see Figure A2. The values of the parameters that were changed to produce the graph are given in Table A2. As long as the curves in the graph are above the ordinate value 1 (marked by a black horizontal line in the graph), the use of the two simplifications is reasonable. In Figure A2 we can see that this is the case for values of truncation factor ν≥0.5. This means that for most practical scenarios (e.g., dazzling of camera systems used for surveillance or in unmanned vehicles at larger distances), simplification S1 and simplification S2 can be applied.

**Table A1:** Values of the parameters that were varied in order to create the plot of Figure A2.

Parameter	Values
λ	400 nm, 550 nm, 700 nm, 1000 nm
f	10 mm, 50 mm, 100 mm
F	1.0, 8.0, 22.0
s	−1, −2, −3
$b_{0}$	0.01, 0.1, 1.0, 10.0
l	10^-2^, 10^-3^, 10^-4^
$N_{\mathrm{ss}}$	2, 10, 20

### Appendix B.2. Applicability of Simplification S3

Urey approximates the diffraction pattern of a truncated Gaussian beam $E_{\mathrm{TrG}}\;\left(r\right)$ (see Equation (14)) by a closed-form expression (Equation (13) of reference [31]), which can be rewritten using the naming conventions of this publication as:

(A2) $$E_{\mathrm{TrG}}\approx E_{\mathrm{env}}\left(r\right)\cdot \sin^{2}\left(\frac{\pi r}{\lambda F}-q\right)$$

$E_{\mathrm{env}}\;\left(r\right)$ is given by Equation (16) and q is a phase factor, which depends itself on r and ν. The estimation of the envelope $E_{\mathrm{env}}\;\left(r\right)$ and the mean $E_{\mathrm{mean}}\;\left(r\right)$ of the diffraction irradiance is based on this expression. According to Urey “the approximate formulas work well beyond the second zero crossing of the function”. The zero crossings can be calculated by solving

(A3) $$\frac{\pi r_{z,n}}{\lambda F}-q=n\cdot \pi$$

using n=2, which leads to

(A4) $$r_{z,2}=\left(2+\frac{q}{\pi }\right)\lambda F.$$

Urey states some values of q for radial coordinates beyond the second zero crossing:

(A5) $$q\left(\nu =0.5\right)=1.04\;q\left(\nu =0.66\right)=0.93\;q\left(\nu =1\right)=0.84\;q\left(\nu \to \infty \right)=0.77$$

The values of q stated in Equation (A5) could be described by the following equation, which would allow the interpolation for different values of ν:

(A6) $$q\left(\nu \right)=0.0713\cdot \nu^{-1.924}+0.77$$

Although I do not know if the extrapolation of q using Equation (A6) is valid, I will use it here to calculate the location of the second zero crossing for extreme values of λ=1000 nm, F=22 and ν=0.1:

$$q\left(\nu =0.1\right)=0.0713\cdot 0.1^{-1.924}+0.77=6.76\;r_{z,2}=\left(2+\frac{6.76}{\pi }\right)\lambda F=4.15\lambda F\approx 91\;µm$$

For typical imaging sensors, this radius would correspond to 15–30 pixels.

I defined the Maximum Dazzle Exposure for a Sensor MDE_S_ in terms of a certain fraction ϵ of the sensor’s FOV that is dazzled (see Section 4.2). According to the preceding result, a reasonable value of MDE_S_ can be calculated for dazzle spots with a radius larger than ~100 µm. Using Equation (46) and Equation (47), this leads to

(A7) $$\Theta_{\epsilon }\cdot f=\epsilon \cdot \frac{FOV}{2}\cdot f=\epsilon \cdot \max\left(N_{\mathrm{col}},\;N_{\mathrm{row}}\right)\cdot \frac{p}{2}>100\;µm$$

and then to

(A8) $$\epsilon >\frac{200\;µm}{\max\left(N_{\mathrm{col}},\;N_{\mathrm{row}}\right)\cdot p}.$$

As an example, for an imaging sensor with 1000 pixel along the long edge and a pixel size of 3 µm, the value of ϵ should be chosen larger than 7 percent for the calculation of the MDE_S_.

## Appendix C. Spot Size Calculations

In Section 6.2, calculated dazzle spot sizes are compared to measurement data in visualized form. The detailed results of the calculations and the data analysis for Setup 1 can be found in Table A2 and Table A3 for the laser wavelengths of 488 nm and 640 nm, respectively. Table A4 presents the results for Setup 2 and the laser wavelength of 640 nm. The results corresponding to the camera images shown in Figure 12; Figure 13 are printed in bold face.

In all tables, there may occur cells containing “n.m.” (not measurable) or empty cells. This is because the dazzle spot in the camera images exceeded the edges of the image. Thus, the counting of overexposed pixels underestimates the real dazzle spot size and does not give reliable results.

**Table A2:** Calculated and measured dazzle spot size using Setup 1 (see Section 6.2) for a laser wavelength of 488 nm. Abbreviation: n.m.: not measurable.

$E_{\mathrm{laser}}\;(µW/{\mathrm{cm}}^{2})$	$P_{\mathrm{in}}\;(µW)$	Calculated Dazzle Spot Radius (pixel)			Measured Dazzle Spot Radius (pixel)	Dazzle Level ϵ	MDE_S_ (µW/cm^2^)
		$\Theta_{\mathrm{dazzle},d}$	$\Theta_{\mathrm{dazzle},s}.$	$\Theta_{\mathrm{num}}$			
2.94 × 10^−04^	7.06 × 10^−04^	2	0	2	2	0.004	1.40 × 10^−04^
3.38 × 10^−03^	8.11 × 10^−03^	5	0	5	3	0.009	1.33 × 10^−03^
4.99 × 10^−02^	0.120	11	0	12	8	0.02	1.60 × 10^−02^
**0.151**	**0.362**	**16**	**0**	**19**	**17**	**0.04**	**0.109**
0.245	0.587	19	7	25	18	0.05	0.122
0.705	1.69	27	34	41	32	0.08	0.410
1.77	4.25	37	56	63	46	0.12	0.882
**3.97**	**9.52**	**49**	**82**	**89**	**72**	**0.19**	**2.41**
7.91	19.0	61	111	118	103	0.27	5.68
9.96	23.9	66	122	130	115	0.31	7.44
12.5	30.1	71	135	142	131	0.35	10.3
15.8	37.9	77	148	156	152	0.40	14.8
25.0	60.1	90	179	188	204	0.54	31.0
31.5	75.7	97	197	206	232	0.62	42.8
39.7	95.2	105	216	225	n.m.	n.m.	-
**51.1**	**123**	**114**	**239**	**249**	**n.m.**	**n.m.**	**-**
60.0	144	120	256	266	n.m.	n.m.	-
70.5	169	127	273	283	n.m.	n.m.	-
99.6	239	143	314	325	n.m.	n.m.	-
125	301	154	344	356	n.m.	n.m.	-
158	379	166	377	390	n.m.	n.m.	-
379	910	223	536	551	n.m.	n.m.	-
488	1170	242	594	609	n.m.	n.m.	-
674	1620	270	675	692	n.m.	n.m.	-

**Table A3:** Calculated and measured dazzle spot size using Setup 1 (see Section 6.2) for a laser wavelength of 640 nm. Abbreviation: n.m.: not measurable.

$E_{\mathrm{laser}}\;(µW/{\mathrm{cm}}^{2})$	$P_{\mathrm{in}}\;(µW)$	Calculated Dazzle Spot Radius (pixel)			Measured Dazzle Spot Radius (pixel)	Dazzle Level ϵ	MDE_S_ (µW/cm^2^)
		$\Theta_{\mathrm{dazzle},d}$	$\Theta_{\mathrm{dazzle},s}.$	$\Theta_{\mathrm{num}}$			
5.45 × 10^−04^	1.31 × 10^−03^	3	0	3	3	0.008	4.44 × 10^−04^
5.20 × 10^−03^	1.25 × 10^−02^	7	0	7	5	0.01	1.96 × 10^−03^
5.97 × 10^−02^	0.143	15	0	15	13	0.03	3.53 × 10^−02^
**0.161**	**0.386**	**21**	**0**	**22**	**20**	**0.05**	**0.119**
0.255	0.611	24	0	27	22	0.06	0.149
0.611	1.47	32	17	40	36	0.09	0.479
1.47	3.52	43	44	59	53	0.14	1.16
2.67	6.40	52	62	76	73	0.19	2.40
**5.32**	**12.8**	**66**	**88**	**102**	**104**	**0.28**	**5.64**
6.40	15.4	70	96	110	113	0.30	6.85
8.06	19.3	76	106	120	126	0.33	8.96
10.1	24.3	82	118	132	151	0.40	14.1
16.1	38.6	95	143	159	196	0.52	27.4
20.2	48.6	103	158	174	223	0.59	37.91
24.9	59.7	110	172	189	n.m.	n.m.	-
31.3	75.2	119	190	207	n.m.	n.m.	-
39.5	94.7	129	209	227	n.m.	n.m.	-
**46.4**	**111**	**136**	**223**	**241**	**n.m.**	**n.m.**	**-**
59.7	143	148	247	267	n.m.	n.m.	-
75.2	180	159	272	292	n.m.	n.m.	-
94.6	227	172	298	320	n.m.	n.m.	-
227	545	230	425	450	n.m.	n.m.	-
286	686	249	466	493	n.m.	n.m.	-
423	1010	283	546	574	n.m.	n.m.	-

**Table A4:** Calculated and measured dazzle spot size using Setup 2 (see Section 6.2) for a laser wavelength of 640 nm. Abbreviation: n.m.: not measurable.

$E_{\mathrm{laser}}\;(µW/{\mathrm{cm}}^{2})$	$P_{\mathrm{in}}\;(µW)$	Calculated Dazzle Spot Radius (pixel)			Measured Dazzle Spot Radius (pixel)	Dazzle Level ϵ	MDE_S_ (µW/cm^2^)
		$\Theta_{\mathrm{dazzle},d}$	$\Theta_{\mathrm{dazzle},s}.$	$\Theta_{\mathrm{num}}$			
5.18 × 10^−04^	7.82 × 10^−04^	2	0	2	5	0.007	4.42 × 10^−03^
4.94 × 10^−03^	7.47 × 10^−03^	5	0	5	8	0.01	2.17 × 10^−02^
5.67 × 10^−02^	8.58 × 10^−02^	11	0	11	14	0.02	9.80 × 10^−02^
0.153	0.231	16	0	16	17	0.02	0.188
**0.242**	**0.366**	**18**	**0**	**19**	**19**	**0.03**	**0.257**
0.581	0.878	25	0	26	26	0.04	0.603
1.39	2.11	33	0	37	43	0.06	1.97
2.54	3.83	40	9	48	57	0.08	3.89
5.06	7.65	50	40	64	75	0.10	7.21
6.08	9.19	54	47	70	80	0.11	8.45
7.65	11.6	58	55	77	87	0.12	10.4
**9.64**	**14.6**	**63**	**64**	**85**	**94**	**0.13**	**12.4**
15.3	23.1	73	82	103	111	0.15	18.4
19.2	29.1	79	92	113	122	0.17	23.5
23.7	35.8	84	102	123	130	0.18	27.3
29.8	45.0	91	114	135	149	0.20	38.6
37.5	56.7	98	126	148	165	0.23	49.8
44.1	66.6	104	136	157	181	0.25	63.2
56.7	85.8	113	152	174	204	0.28	85.0
**71.4**	**108**	**122**	**168**	**191**	**236**	**0.32**	**124**
89.9	136	132	185	209	276	0.38	185
216	326	176	266	294	459	0.63	680
272	411	191	293	322	n.m.	n.m.	-
402	607	217	343	375	n.m.	n.m.	-
