# Correction: Ritt, G. Laser Safety Calculations for Imaging Sensors. *Sensors* 2019, *19*, 3765

**DOI:** 10.3390/s21061959

**Published:** 2021-03-15

**Authors:** Gunnar Ritt

**Affiliations:** Fraunhofer IOSB, Gutleuthausstr. 1, 76275 Ettlingen, Germany; gunnar.ritt@iosb.fraunhofer.de

The author wishes to make the following corrections to the paper [1]:

On page 5, Section 3.1, “The peak irradiance $E_{0}$ (W) of the diffraction pattern at the focal plane is given by…”, should be updated to, “The peak irradiance $E_{0}$ (W/m^2^) of the diffraction pattern at the focal plane is given by…”.

On page 20, Section 4.4, the term $P_{\mathrm{laser}}T\lambda F/\pi^{3}f^{3}$ is missing in Equation (59). The correct equation would be:

(59) $$E_{d}\left(\Theta_{\mathrm{dazzle},d}\right)\;\approx \;E_{\mathrm{mean}}\left(\Theta_{\mathrm{dazzle},d}\right)\;=\;\frac{P_{\mathrm{laser}}T\lambda F}{\pi^{3}f^{3}}\cdot \frac{1}{\Theta_{\mathrm{dazzle},d}{}^{3}}\cdot \frac{2}{\nu^{2}}\exp\left(-\frac{2}{\nu^{2}}\right)\;=\;E_{\mathrm{sat}}$$

On page 20, Section 4.4, the parameter $\nu^{*}$ is missing in Equations (60) and (62). The correct equations would be:

(60) $$E_{s}\left(\Theta_{\mathrm{dazzle},s}\right)\;=\;\frac{P_{\mathrm{laser}}TN_{\mathrm{ss}}b_{0}}{f^{2}}\frac{1}{{\left(v^{*}\right)}^{2}}{\left[1\;+\;\frac{1}{{\left(v^{*}\right)}^{2}}{\left(\frac{\Theta_{\mathrm{dazzle},s}}{l}\right)}^{2}\right]}^{\frac{s}{2}}\cdot \left(1\;-\;\exp\left(-\frac{2}{\nu^{2}}\right)\right)\;=\;E_{\mathrm{sat}}$$

(62) $$\Theta_{\mathrm{dazzle},s}\;=\;v^{*}\cdot l\cdot \sqrt{{\left(\frac{E_{\mathrm{sat}}}{P_{\mathrm{laser}}T}\cdot \frac{f^{2}{\left(v^{*}\right)}^{2}}{N_{\mathrm{ss}}b_{0}}\cdot \frac{1}{\left(1\;-\;\exp\left(-\frac{2}{\nu^{2}}\right)\right)}\right)}^{\frac{2}{s}}\;-\;1}$$

The author apologizes for any inconvenience caused and states that the scientific conclusions are unaffected. The original article has been updated.
